# Cannabinoids—Characteristics and Potential for Use in Food Production

**DOI:** 10.3390/molecules26216723

**Published:** 2021-11-06

**Authors:** Joanna Kanabus, Marcin Bryła, Marek Roszko, Marta Modrzewska, Adam Pierzgalski

**Affiliations:** Department of Food Safety and Chemical Analysis, Prof. Wacław Dąbrowski Institute of Agricultural and Food Biotechnology—State Research Institute, Rakowiecka 36, 02-532 Warsaw, Poland; marcin.bryla@ibprs.pl (M.B.); marek.roszko@ibprs.pl (M.R.); marta.modrzewska@ibprs.pl (M.M.); adam.pierzgalski@ibprs.pl (A.P.)

**Keywords:** bioactive compound, cannabis, cannabidiol, tetrahydrocannabinol, food safety, food analysis, cannabinoids, hemp food

## Abstract

Scientific demonstrations of the beneficial effects of non-psychoactive cannabinoids on the human body have increased the interest in foods containing hemp components. This review systematizes the latest discoveries relating to the characteristics of cannabinoids from *Cannabis sativa* L. var. *sativa*, it also presents a characterization of the mentioned plant. In this review, we present data on the opportunities and limitations of cannabinoids in food production. This article systematizes the data on the legal aspects, mainly the limits of Δ9-THC in food, the most popular analytical techniques (LC-MS and GC-MS) applied to assay cannabinoids in finished products, and the available data on the stability of cannabinoids during heating, storage, and access to light and oxygen. This may constitute a major challenge to their common use in food processing, as well as the potential formation of undesirable degradation products. Hemp-containing foods have great potential to become commercially popular among functional foods, provided that our understanding of cannabinoid stability in different food matrices and cannabinoid interactions with particular food ingredients are expanded. There remains a need for more data on the effects of technological processes and storage on cannabinoid degradation.

## 1. Introduction

*Cannabis sativa* L. is one of the oldest cultivated plants on the planet. Initially, it was used by humans as a source of roughage in animal fodder and as a textile fiber; over time, it was used as a source of food and medicine [1,2]. The plant contains bioactive compounds called cannabinoids [3]. The medicinal use of hemp in Europe dates to the 13th century, however their anticonvulsant, analgesic, and antiemetic properties were not confirmed until the 19th century [3,4]. In Europe, at the end of the 1950s, Russia and Italy were the leading countries in land area used for the cultivation of hemp, as well as in the quality of the products obtained [5,6]. However, after it was observed that hemp’s Δ9-THC induces psychotropic effects, the awareness of its adverse effects on the human body increased, and because of this, numerous countries ceased to grow the plant and use its seeds and flowers in food production. After many years, hemp has gained popularity owing to its low soil and hydrological requirements; it can be grown on almost every soil, regardless of climatic conditions and without special fertilizers. Thanks to these advantages, hemp is becoming a symbol of sustainable farming. Non-narcotic hemp varieties have also been found to have positive effects in the treatment of many diseases [3,6].

Canada is one of the first countries to allow industrial hemp cultivation and remains a major distributor in its production and export, including in food [6,7]. The European Union is the second largest producer of *Cannabis sativa* L. in the world, with centers in France, the Netherlands, Lithuania, and Romania [7,8]. For centuries, *C*. *sativa* L. has been used as an invaluable source of plant fiber. In the last decade, there has been a growing interest in hemp seeds, which contain compounds that have beneficial effects on the human body and considerable nutritional value [6]. Considering the significant interest of scientists in the potential health benefits of cannabinoids from *Cannabis sativa* L. var. *sativa* in food production, we aim to summarize the latest knowledge in terms of the characteristics of the plant, cannabinoids, and assess the potential of cannabinoids for use in food. Documentation was performed via the Scopus, ScienceDirect, and Google Scholar databases, mostly selecting publications after the year 2010.

## 2. *Cannabis sativa* L. var. *sativa*—Classification and Characteristics

*Cannabis sativa* L. belongs to the order Urticales and the family Cannabaceae. It is an annual plant that grows in the Northern Hemisphere in moderate climates [9]. The exact areas in which hemp originally grew are not known because the plant has spread all over the world and has been evolving for centuries [1,2,3,4,5,6,7,8,9]. There are reports of the cultivation and use of *Cannabis sativa* L. in the Neolithic period. The first documented evidence of the pharmaceutical use of the plant was found in cave artefacts dating to ca. 700 BCE. More detailed analyses suggest that *Cannabis sativa* L. may have originated in Central Asia, and then spread to Mediterranean countries, Eastern and Central European countries, and, in particular, to Afghanistan and Pakistan. There are reports of two other centers of species diversity of *Cannabis sativa* L., these being Hindustani and European–Siberian [8,10].

Hemp is characterized by a small number of broadly spaced branches and long, palmately compound leaves. On one branch, 3–13 leaves are present [9]. Hemp is mostly diclinous. Female plants are frost-resistant and grown in greenhouses or in countries without low temperatures where they can survive for several years. It is important to say that the monoecious hemp, thanks to its better usability, has largely replaced the dioecious hemp in Europe. *Cannabis sativa* L. reaches a height of 1–5 m, depending on the environment. Usually, its vegetative period lasts for 3–4 months [1,9,11,12,13,14].

Hemp’s considerable genetic variability makes its taxonomic classification more difficult. Studies comparing the content of chemical compounds between groups of cultivated and wild plants have led to contradictory interpretations and multiple hemp classifications. At present, 750 natural compounds have been identified in hemp that represent different chemical classes, indicating a very complex phytochemistry [2,13]. Its primary metabolites include amino acids, fatty acids, and steroids. Its secondary metabolites include phytocannabinoids, flavonoids, terpenoids, lignans, and alkaloids [15]. Phytocannabinoids are the best-studied hemp compounds. The discovery and understanding of the biosynthetic pathway of phytocannabinoids are crucial to show that the concentration of each compound present in the plant is determined genetically because different genotypes are characterized by different cannabinoid profiles [6].

The number of species in the genus *Cannabis* has been disputed by taxonomists for a long time. There is no clearly defined nomenclature in literature for *Cannabis*. The simplest division of the plants in the genus is into three separate species: *Cannabis sativa* (fiber hemp), *Cannabis indica* (Indian hemp), and *Cannabis ruderalis* (considered to be a wild form) [1,9]. An alternative classification distinguishes *Cannabis* chemotypes based on cannabinoid content. Chemotype I is medicinal and contains large amounts of psychoactive Δ9-THC (Δ9-tetrahydrocannabinol). Chemotype II has intermediate properties between medicinal and fiber hemps. Chemotypes III and IV are fibrous and contain large amounts of non-psychoactive cannabinoids, with relatively low amounts of psychoactive compounds. The last group (chemotype V) is fibrous and contains no cannabinoids [1]. Farinon et al. [6] categorized only three chemotypes based on Δ9-THC and cannabidiol (CBD) content. These are chemotype I, with a low ratio of CBD to Δ9-THC (more than 0.2% Δ9-THC content in plant dry matter during the flowering period, grown for recreational/narcotic purposes); chemotype II, with the two main cannabinoids, CBD and Δ9-THC, at similar concentrations, but usually with slightly higher CBD content (for medicinal purposes); and chemotype III, characterized by a high CBD content, with Δ9-THC content not exceeding 0.2% of plant dry matter (used for industrial purposes and in food). Currently, all the varieties are treated as one diverse species, *Cannabis sativa* L., with varieties being *C. sativa* L. var. *sativa*, *C. sativa* L. var. *indica*, and *C. sativa* L. var. *ruderalis* [16]. This classification has also been used in the work of.

*C. sativa* L. produces a small fruit referred to as achenes in botany, but commonly referred to as “seeds”. The pericarp is a protective layer for the grain contained in it. The fruit consists of an embryo and mainly two seed leaves that are rich in oils, carbohydrates, and proteins [17]. *C. sativa* L. var. *sativa* seeds are characterized by a high fiber (27–36 g/100 g), fat (25–35 g/100 g), protein (21–28 g/100 g), and carbohydrate (20–30 g/100 g) content. The chemical composition of seeds also includes fatty acid esters, amides, amines, phytosterols, terpenes, phenolic compounds, and cannabinoids [1,2,6,17,18]. They further contain phosphorus, potassium, magnesium, sulphur, calcium, iron, and zinc, as well as vitamins A, C, and E [2,6]. The unique nutritional value of hemp seeds stems from their high content of essential unsaturated fatty acids (approximately 80% of total fatty acid content) [17]. The ratio of omega-6 to omega-3 fatty acids in hemp seed oil is usually 2:1 or 3:1, which is considered optimal for human health [19]. Linoleic acid accounts for more than half of the total fatty acid content (LA, 18:2, n-6). The remaining fatty acids include linolenic acid (ALA, 18:3, n-3), oleic acid (OA, 18:1, n-9), palmitic acid (PA, 16:0), and γ-linolenic acid (GLA, 18:3, n-6) [18]. It should be remembered that maintaining the appropriate ratio of omega-6 to omega-3 acids depends on a used method of oil extraction (cold pressing is recommended), the origin of the variety, and the type of seeds [20]. Hemp seed-derived proteins include albumin, globular protein, and estidine, the consumption of which has a beneficial effect on regulating human metabolism. Estidine is the most abundant component, accounting for approximately 82% of total protein in hemp seeds [6]. Estidine has a high biological value because its structure is similar to that of globulins present in blood serum, which means that these compounds can be used for the biosynthesis of immunoglobulins, hormones, and enzymes [21]. This protein contains all the essential amino acids [22]. The amino acid profile of hemp seed protein is comparable to that of chicken eggs, but also to that of soybeans, which feature high concentrations of arginine, glycine, and histidine [22,23]. Callaway [23] confirmed these nutritional properties of hemp seeds by finding considerable amounts of protein and essential unsaturated fatty acids. Despite the positive effect of hemp seed-derived protein, attention needs to be given to the antinutritional compounds present in hemp seeds. These contain phytic acid and trypsin inhibitors, which can negatively affect human digestive function [22].

The characteristic scent and flavor of hemp are attributed to terpenes. Both mono- and sesquiterpenes have been isolated from hemp roots and aerial parts. At least 200 different terpenes of the 20,000 known in nature have been found. In most hemp varieties, the most common is monoterpene myrcene as well as sesquiterpenes such as β-caryophyllene and α-humulene. Among the remaining terpenes found in *Cannabis sativa* L. var. *sativa* are α-pinene, limonene, linalool, bisabolol, and (E)-β-farnesene [24,25]. According to some studies, hemp terpenes have antidepressant, anti-inflammatory, and anxiolytic effects. They participate in photosynthesis and have a protective role in plants [2]. André et al. [11] demonstrated that, as the flowers develop, their content of sesquiterpenes decreases, while their content of monoterpenes increases. At the end of the flowering period, monoterpenes account for more than 50% of terpenes in flowers. It is difficult to study the precise content of terpenes in plants because, depending on geographic location, terpene profiles can differ within the same variety [1,8,11,25]. Ingallina et al. [26] showed that each of the analyzed species is characterized by a specific terpenoid profile in the inflorescences. The caryophyllene E, caryophyllene oxide, and humulene were always present, and the content of the compounds was variable depending on the variety and the growth period.

Fibrous hemp inflorescences, as a by-product in the textile industry, are a source of polyphenol compounds with demonstrated health-promoting properties [25]. Flavonoids constitute the largest class of polyphenols and have been divided into six main subclasses: flavones, flavonols, flavanones, flavanols, isoflavones, and anthocyanidins [2,25]. Flavonoids make up about 10% of the total compounds present in hemp. The group of flavonoids isolated from flowers, leaves, and pollen includes the O-glycoside aglycone derivatives apigenin, luteolin, orientin, kaempferol, and quercetin, but also cannflavins A and B, which are methylated isoprenoid flavones unique to hemp [2,15]. In tests conducted by André et al. [11], the flavonoid content in hemp inflorescences was determined depending on flowering time. It was observed that the content of phenolic compounds decreased as the flowers developed, with the highest content reported in flowers collected in the early flowering phase. Ingallina et al. [26] also demonstrated a decrease in the content of flavonoids during inflorescence maturation. Spano et al. [27] showed an increase in the content of polyphenic compounds in the first and second phase of inflorescence maturation. The decrease in the content of this group of compounds took place at the end of maturation. In particular, phenolic acids and flavonoids were present among the high concentration of polyphenolic compounds in the inflorescences. Despite the differences in the concentration of flavonoids depending on the variety, it was found that the female inflorescences mainly contained flavone derivatives (apigenin and luteolin). Male inflorescences contain fewer flavonoids than female inflorescences but are characterized by the presence of two unique flavonol compounds, quercetin-O-sophoroside and kaempferol-O-phosphoroside. Flavonoids can constitute approximately 2.5% of the dry matter of hemp leaves and inflorescences, while in the roots and seeds they are present in negligible amounts [25]. Stilbenoids are another group of phenolic compounds that demonstrate a protective and insect-repelling role in hemp. These compounds are present in the leaves, stems, and cannabis resin. Other secondary metabolites of *Cannabis sativa* L. var. *sativa* are alkaloids that show a wide range of bioactivities. They act as end products of metabolism and as animal repellents. Multiple alkaloids have been isolated from leaves, stems, and seeds, these being choline, nurine, muscarine, and hordenine [2,15,28,29].

*Cannabis sativa* L. var. *indica* is used for marijuana, hash, and hash oil; these are narcotic preparations because they contain high amounts of psychoactive Δ9-THC [30]. *Cannabis sativa* L. var. *indica* is particularly common in Southeast and Central Asia. It can be found in European countries and rarely found in South America, Australia, and Africa [1,9]

According to an EIHA report [8], over the years 2010–2013, the production of the seeds for food purposes in the US increased by 92%. In 2013, the production of flowers and leaves used in medicine, dietary supplements, and oils increased by 3000% compared to 2010. In 2019, the size of the global cannabis market was valued at USD 123.9 billion. The market is expected to grow at an annual growth rate of 14.3% from 2020 to 2027. The liberalization of hemp cultivation (mainly of varieties with low THC content), the potential use of hemp plants in the treatment of chronic diseases, and the use of hemp as a food ingredient are major factors contributing to the growth of the hemp cultivation market [8,30].

## 3. Hemp Cannabinoids

**Figure 1:**
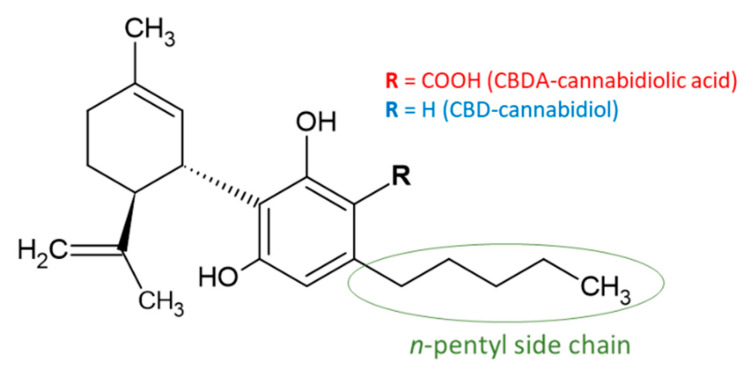
Typical cannabinoid structures (e.g., CBD) differ in carboxylic acid, neutral form, and alkyl side chain [13,16,31].

Cannabinoids (phytocannabinoids) are one of the most important hemp bioactive compounds [1]. They are meroterpenoids with resorcinol cores containing an isoprenyl, alkyl, or aralkyl side chain in the para position. An alkyl side chain is usually composed of an uneven number of carbon atoms. Orcinoids contain one carbon atom, three varinoids, and five carbon atoms. Cannabinoids containing an even number of carbon atoms in the side chain occur very rarely in plants. *Cannabis sativa* L. usually produces alkyl cannabinoids that are characterized by a monoterpene-isoprenyl (C10) moiety and a pentyl (C5) side chain [13,31]. The ring structure originates from geranyl pyrophosphate [16]. A typical cannabinoid structure is shown in Figure 1.

Phytocannabinoids are synthesized in the glandular trichomes of the plant, primarily found in female inflorescences. Hemp seeds usually contain very small amounts of cannabinoids or none whatsoever. When the glandular trichomes crack, such as due to the impact of higher temperatures, a viscous coating (resin) forms on the surface of the plant, which covers all the elements of inflorescence elements, including the seeds. Resin cannabinoids may be considered as a seed contaminant [17,31]. Based on chromatographic analysis, cannabinoids are present at much lower concentrations in stems, pollen, and roots [15]. Since the beginning of studies relating to the cannabinoids present in *Cannabis sativa* L., more than 100 cannabinoids have been identified, some of them having a beneficial effect on the functioning of the human body [3].

Selected cannabinoids isolated from *Cannabis sativa* L. are presented in Table 1. Currently, it is believed that cannabinoids are initially synthesized in an acidic form and then, only as a result of decarboxylation and/or the appropriate enzymes, they are converted into their neutral forms [31,32].

**Figure 2:**
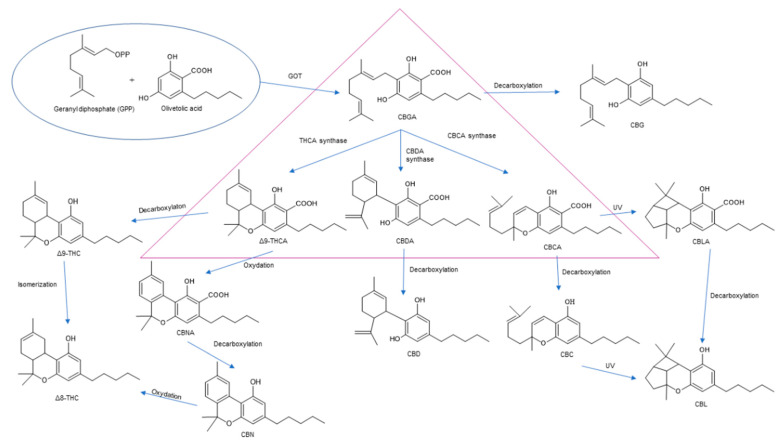
Biosynthesis pathways of selected cannabinoids [13,17,33,39].

Multiple studies have shown that the first step in cannabinoid biosynthesis is the formation of olivetolic acid, but its biosynthetic pathway has not yet been fully elucidated [16,32,36]. The biosynthesis of cannabigerolic acid (CBGA) occurs in the presence of olivetolic acid precursors and geranyl pyrophosphate with the participation of prenylase geranyl diphosphate:olivetolate geranyltransferase (GOT). This enzyme catalyzes the first step in the synthesis of cannabinoids. This reaction produces CBGA and cannabigerovanic acid; these are also precursors for the synthesis of many other cannabinoids that are produced [3,16,17,22,32,37]. Subsequently, after the isoprenylation step, oxidative cyclase becomes active, which generates further reactions through specific enzymes. CBGA transformations catalyzed by tetrahydrocannabinolic acid synthase lead to the formation of tetrahydrocannabinolic acid (Δ9-THCA). However, as a result of the activity of cannabidiol acid synthase, CBGA transformation leads to the formation of cannabidiolic acid (CBDA), while the conversion of CBGA catalyzed by cannabichromenic acid synthase allows for the formation of cannabichromenic acid (CBCA). Then, as a result of the decarboxylation of these compounds, many products can be formed: Δ9-tetrahydrocannabinol (Δ9-THC), cannabidiol (CBD), and cannabichromen (CBC). The same reaction pathway is observed in the biosynthesis of the remaining cannabinoid acids from cannabigerovarinic acid [6,17,22,32,38]. The biosynthesis pathways of selected cannabinoids are illustrated in Figure 2.

The best-known cannabinoids that occur in *Cannabis sativa* L. are Δ9-THC and CBD. The global interest in CBD is growing due to its demonstrated analgesic, anti-inflammatory, and anxiolytic properties [1,39,40,41]. The chemical structure of CBD was determined by Mechoulam and Shvo in 1963 [42], with Δ9-THC being an isomer of this compound. The properties of selected neutral cannabinoids are listed in Table 2.

The strongest psychoactive cannabinoid, Δ9-THC, belongs to a group of compounds that are subject to very strict international control. This was the first phytocannabinoid isolated from *Cannabis sativa* L. [54,56,57] and is a degradation product of Δ9-THCA [58]. It occurs in the highest concentrations in *Cannabis sativa* L. var. *indica* and interacts with the signaling system of the endocannabinoid cell in the human body. It plays a regulatory role in key functions including memory, homeostasis, appetite, and reproduction [2]. The Δ9-THC binds and activates the CB1 receptors of the endocannabinoid system, which are mainly present in the central nervous system as well as in the digestive tract, liver, fatty tissue, kidneys, muscles, and heart. Activation of these receptors inhibits the release of hormones (prolactin, estradiol, and progesterone) and neurotransmitters (acetylcholine, dopamine, and serotonin). The endocannabinoid system also includes CB2 receptors that are present on the surface of immune cells. Their activation stimulates the release of anti-inflammatory cytokines [51,52]. The most common route of administration of Δ9-THC to humans is the oral route, from there it is easily distributed into vascularized tissues such as the liver and lungs [1,25,39,40,41]. As a drug, Δ9-THC is used to induce appetite and for its antiemetic properties for people undergoing chemotherapy, but it is also used to improve sleep [31,53]. Δ8- Tetrahydrocannabinol (Δ8-THC) is an isomer of Δ9-THC, which differs by the location of the double bond. This compound also demonstrates psychoactive properties but is much more chemically stable than Δ9-THC [44].

Phytocannabinoids with confirmed psychoactive properties include Δ8-THC, which shows a slightly weaker effect on cannabinoid receptors. Its anti-glaucoma properties have also been confirmed, which is due to the compound’s ability to affect the intraocular pressure in humans [31,59]. Moreover, cannabinol (CBN), which remains a poorly studied compound, shows tenfold weaker psychoactive properties than Δ9-THC [25]. This product of Δ9-THCA degradation is produced when the acid is heated. Its acidic form is present in the entire *Cannabis sativa* L. plant. CBN binds cannabinoid receptors, showing higher affinity for CB2 receptors, and it is a weak agonist for CB1 receptors [44].

CBD is one of the best-studied cannabinoids present in *Cannabis sativa* L. This compound does not have narcotic effects; therefore, it is highly likely that it can be used therapeutically [25,31]. From in vitro studies, CBD is characterized by a weak antagonism for CB1 and CB2 receptors [44,46]. Afrin et al. [47] and Kis et al. [48] confirmed in their studies the benefits of CBD use in patients with lung cancer, breast cancer, and leukaemia. CBD is also noted for its anticonvulsant, anxiolytic, and anti-rheumatoid arthritis properties. To reduce the psychotic symptoms induced by Δ9-THC, CBD can be used to eliminate the negative effects of Δ9-THC on hippocampus-dependent memory [31,44].

In addition to CBD, confirmed non-psychoactive properties have been attributed to cannabigerol (CBG), one of the major cannabinoids produced by *Cannabis sativa* L., which is present in much lower amounts than Δ9-THC and CBD. It was the first compound to be purified from *Cannabis sativa* L. resin. The structural property of this compound is the presence of a linear isoprenyl residue. Hemp varieties with significantly higher CBG content are referred to as type IV cannabis (containing significant amounts of non-psychoactive cannabinoids). Due to the lack of narcotic effects, CBG is gaining popularity, with varieties being developed that produce larger amounts of CBG and CBGA [16,25,32]. The acidic form of CBG and CBGA is a precursor for the biosynthesis of other important cannabinoids. CBG has a low affinity for CB1 and CB2 cannabinoid receptors; however, it inhibits the functioning of the endocannabinoid system [43,44,57].

CBC is another compound that does not have psychoactive effects in humans. It occurs in dried hemp material in considerable amounts because its synthesis relies on the decarboxylation of CBCA induced by heating. Although it is present in all *Cannabis* varieties, its properties have not been fully elucidated. The isoprene residue of the compound is oxidatively bound to the resorcinol ring. In many varieties of *Cannabis*, the presence of CBC is associated with the presence of Δ9-THC. CBC is only one of the major cannabinoids, which is characterized by blue fluorescence under UV light [25,32]. Δ9-Tetrahydrocannabivarin (Δ9-THCV) is a homologue of Δ9-THC, whose side chains contribute to effects other than Δ9-THC, which makes it a phytocannabinoid with non-psychoactive properties. Considerably higher amounts of Δ9-THCV are present in fibrous hemp than in *Cannabis sativa* L. var. *indica*. Δ9-THCV is a partial agonist of the CB2 receptor, whose activity has been measured both in vitro and in vivo [25,44,58].

Although more than 100 phytocannabinoids present in hemp have already been discovered, most of them have not been fully characterized yet [35]. We know very little about cannabidivarin (CBDV), cannabivarin, cannabielsoin, cannabicyclol, cannabitriol, and cannabitriol. CBDV is a CBD derivative that differs from cannabidiol only in that it has a shorter side chain. It has a very weak affinity for CB1 and CB2 receptors. The psychoactive properties of this compound have not yet been confirmed [25,44,60]. Cannabivarin, also known as cannabivarol, is a CBN analogue with a shorter side chain. It is present in small amounts in *Cannabis sativa* L. and is rarely found in fresh plants but mainly present in dried hemp. This compound can be obtained as a result of Δ9-THCV oxidation [25,44]. CBD can be produced by the photooxidation of CBDA and CBD [32,44]. Cannabitriol is produced by heating CBC; its biosynthesis begins when the oxidation of Δ9-THC begins. The affinity of these compounds for the CB1 and CB2 cannabinoid receptors has not yet been described [44].

In 2019 [61], new phytocannabinoids—cannabiphorol and Δ9-tetrahydrocannabiphorol—were isolated from hemp. These compounds have seven carbon alkyl chains. They are the first phytocannabinoids that contain more than five carbon atoms in the chain, which is unlike most of the cannabinoid compounds isolated from *Cannabis sativa* L. Based on in vitro studies, the capacity of tetrahydrocannabiphorol to bind to the CB1 receptor is 30 times higher than that of Δ9-THC [61].

Although cannabinoids have gained popularity for their beneficial effects in humans, which have been confirmed in many cases, reports on their antimicrobial properties should also be noted but are still rare and often overlooked. Appendino et al. [62] examined the antimicrobial properties of the major cannabinoids CBD, CBC, CBG, Δ9-THC, and CBN, showing that each had activity against methicillin-resistant *Staphylococcus aureus*. Ali et al. [63] confirmed that hemp extracts have antimicrobial effects. Extracts containing mainly Δ9-THC and CBD are active against Gram-positive bacteria (*Bacillus subtilis* and *Staphylococcus aureus*), while the remaining cannabinoids show bactericidal effects on Gram-negative bacteria (*Escherichia coli* and *Pseudomonas aeruginosa*). For fungi, hemp water and acetone extracts were compared. The acetone extract showed higher antimicrobial activity against the bacteria *Pseudomonas aeruginosa* and *Vibrio cholerae*, as well as against the fungi *Cryptococcus neoformans* and *Candida albicans*. This could indicate that acetone extracts contain more extracted cannabinoids than water extracts [64]. As confirmed by Ali et al. [63], numerous fungi can metabolise cannabinoids without adversely affecting *Aspergillus niger*. Iseppi et al. [65] concluded that hemp essential oils show promise of being antibacterial against Gram-positive bacteria; unfortunately, they were ineffective against Gram-negative bacteria. The oils with a mixture of bioactive substances, including cannabinoids, showed a stronger antibacterial effect, which was probably due to the synergistic interactions between the compounds present in the oils. They found that hemp essential oils inhibited bacterial proliferation, which can ultimately improve the microbiological quality of finished products containing hemp extracts, oils, or components. A study by Frassinetti et al. [66] showed that *Cannabis sativa* L. seed extract had selective inhibitory activity against pathogenic strains. It also inhibited the production of biofilms by *Staphylococcus aureus*. The study did not specify which compounds (e.g., cannabinoids) present in the extracts were responsible for such effects on bacteria. The use of seed extracts or other components of *Cannabis sativa* L. may be an alternative to other methods used to control microbial growth in finished products available on the food market.

The constantly increasing interest in hemp and its compounds has led to a growing concern about the safety of dietary supplements, dried hemp, and food containing cannabinoids. Over the past decade, many studies on the properties of cannabinoids have opened up new possibilities for the use of cannabinoids other than Δ9-THC and CBD in addressing multiple human health problems. Despite this, many properties of cannabinoids present at lower concentrations than the main cannabinoids have yet to be discovered; the acquisition of this knowledge will allow for the wider use of such an abundant group of compounds in medicine and functional food production.

Cannabinoids may be introduced into the human body through both the respiratory and digestive tracts. For this article, their bioavailability and effects on the human body after ingestion will be reviewed. Despite the constantly growing interest of consumers in cannabinoids, there is still a lack of knowledge about their metabolic and homeostatic effects.

The most popular cannabinoid consumer products are dietary supplements, food and beverages containing hemp extracts or *Cannabis sativa* L. plant components [67]. The consumption of CBD and Δ9-THC causes their bioavailability to decrease (< 20%) because they are degraded in the acidic environment of the stomach and also in the intestines by enzymes [68,69]. The oral administration of cannabinoids leads to an intensive increase in the hepatic metabolism of these compounds. The maximum plasma concentrations of Δ9-THC and CBD after the consumption of hemp products are reached much later (1–2 h after consumption) than after smoking marijuana [70,71]. The application of hemp products to the oral mucous membrane allows for higher plasma concentrations to be achieved faster than ingestion [71]. In medicine, ingestion is the most frequent route of administration for medicinal therapies [70]. It has been demonstrated that Δ9-THC passes through the placenta and can reach the fetus, but concentrations in fetal blood are usually lower than in maternal blood [72]. It was demonstrated that the ingestion of two Δ9-THC doses (0.75 and 1.5 mg) per day was safe and well-tolerated by elderly patients suffering from dementia. Δ9-THC was quickly assimilated in the body of these persons, with maximum plasma concentrations of this compound (0.41 and 1 μg/L at 1 and 2 h following ingestion, respectively) reached rapidly [73]. Cannabinoids are metabolized in the liver, and they take several days to be removed from the body. The half-life of Δ9-THC and CBD after ingestion is very difficult to quantify because it is influenced by the balance between the plasma and the fatty tissue [70,71]. Ingested Δ9-THC is 80–90% excreted in the form of carboxy and hydroxy derivatives, while CBD, which is not metabolized, is excreted in the feces [67,69,70].

According to the available literature, there is no evidence to confirm that the excessive use of cannabinoids (mainly Δ9-THC) may lead to overdose or death, but there are reports of children falling into comas after consuming these compounds [69,74]. For many years, it has been commonly believed that the overuse of *Cannabis sativa* L. leads to addiction. It is presently believed that the most frequent disorders resulting from long-term *Cannabis* use are increased blood pressure, bronchitis, and reduced lung capacity in persons who smoke it. Certain cannabinoids can also affect fertility and the immune system, as well as cause mood problems and anxiety [69].

There are still many unknowns related to the action and/or interactions of most cannabinoids in the human body. It is extremely important to understand the pathways of cannabinoid transformation and degradation after consuming products containing these compounds for their toxicity in order to ensure food safety. Further studies are needed to gain insight into the benefits of using such products and their long-term adverse health effects in humans, which are still unclear, especially for cannabinoids that occur in lower amounts than CBD and Δ9-THC.

## 4. *Cannabis sativa* L. in Food Production—Opportunities and Limitations

In recent years, there has been an increasing consumer interest in hemp seeds and the products that contain them. The growing popularity of such products translates into an ever-growing product range. Presently, the most popular food products are produced from seeds, hemp flour, and hemp oil. [39,44,75]. Products containing cannabinoids are made by adding cannabis flour derived from the seeds as well as cannabinoid oils and/or extracts. The proportion of a given addition affects the final content of these compounds in the finished product, but the content mainly of THC must not exceed permissible limits [44,76,77,78,79].

The concentration of cannabinoids in the oils depends on the variety and the seed cleaning process. Cannabinoids are considered by some to be an impurity in pressed hemp oils [17]. The use of hemp in food processing is extremely difficult due to the differences in the cannabinoid content in plants. A study by Hazekamp and Fischedick [9] showed that nominal cannabinoid concentrations in plants of the same type but in varied geographical locations differed by more than 25%. To mitigate such differences, we can implement strict control over the varieties and their growing methods to ensure greater homogeneity or mix the extracts to ensure the desired homogeneity [9,77,78,79].

Regulations regarding the permitted content of cannabinoids in food products vary around the world. Legal restrictions usually only apply to Δ9-THC and do not include Δ9-THCA, which converts into Δ9-THC after thermal treatment. Although legal limits differ from country to country, the ranges are usually expressed in mg/kg (ppm) of the final product [39]. Examples of legal limits applicable to selected countries are presented in Table 3.

In Europe, *Cannabis sativa* L. varieties can be used in food production wherein the total content of Δ9-THC and Δ9-THCA in blossoming or fruiting plant tips from which the resin has not been removed does not exceed 0.2% of dry matter. [86]. Only seeds from hemp can be used for food purposes. Other hemp parts and their extracts are classified as “novel foods” in accordance with the Regulation of the European Parliament and the Council (EU) 2015/2283 of 25 November 2015. This regulation defines the term as food that had not been traditionally consumed within the EU before 15 May 1997 [87]. This group includes foods with new or modified molecular structures; foods derived from products produced by microorganisms, fungi, or seaweed; and whole plants or substances extracted from them. *Cannabis sativa* L. var. *sativa* seed extracts and their derivative products containing cannabinoids are considered “novel foods” because no history of their consumption has been demonstrated. However, using plant parts other than seeds can be dangerous because the cannabinoid content in different parts of the plant will vary depending on the variety or the growing conditions; this might constitute a threat to human health.

The European Food Safety Authority [88] has established the acute human reference dose (ARfD) for Δ9-THC as 1 μg Δ9-THC/kg. According to the recommendations of the European Commission, there is a need to monitor food made from hemp or containing hemp components in terms of their content of Δ8-THC, CBN, CBD, and Δ9-THCV [89]. Despite the established acceptable limits for Δ9-THC in many products containing cannabinoids, considerable breaches have occurred [90]. Products containing CBD are not explicitly regulated by the EU because CBD is not classified as a controlled substance. Therefore, consumers have no legal guarantee of the quality of a given product [45]. According to studies by Bonn-Miller et al. [91], as much as 69% of the 84 products from different vendors from the US containing CBD were characterized by erroneous labelling, while in 42% of the products, the concentration of this compound did not match the one declared by the manufacturer. In Germany, 67 products containing CBD were analyzed. In 25% of these, the Δ9-THC content exceeded the ARfD [92]. Producers should ensure that products containing cannabinoids are correctly labelled. Research is also necessary to show the changes in these compounds during storage in order to ensure that the consumer is able to consume the product with the declared amount of cannabinoids throughout its shelf-life. Another challenge is that many countries have their own limits for the content of these substances in finished products. The lack of uniformity prevents the distribution of these products beyond the borders of the country in which they were produced [93].

**Figure 3:**
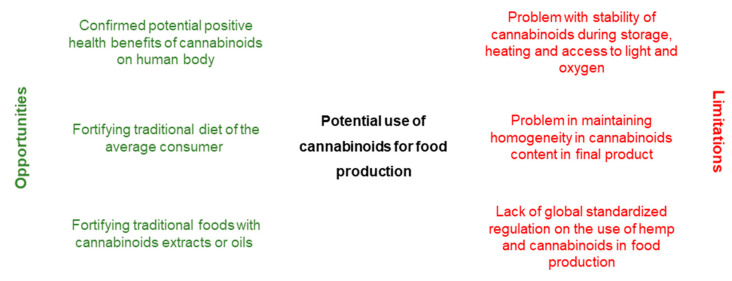
Major opportunities and limitations that may have an impact on the potential use of cannabinoids in food production [94,95,96,97,98,99,100].

Hemp extracts are characterized by a resinous, oily texture and their solubility in organic solvents, fats, and alcohols. The appropriate selection of the form (oil, extract) in which the cannabinoids are to be added to the finished product is important so that they have adequate solubility and do not affect the formulation of the finished product. Extracts containing Δ9-THC and/or CBD usually dissolve in edible oils (e.g., coconut or olive). However, further use of these extracts in processing requires the preparation of an oil and water mixture. Therefore, liquid formulations in which the oil phase is dispersed in the aqueous phase ensure good solubility in water and reduce the susceptibility of the cannabinoids contained in them during oxidation [94,95,96]. Oil–water emulsions containing cannabinoids are used in the production of tinctures, soft capsules, and beverages. This type of emulsion requires surfactants (emulsifiers) such as polysaccharides, proteins, and phospholipids. The choice of emulsifier depends on the emulsion type, oil molecular composition, and ionic strength of the aqueous extract. Solid hemp products are difficult to produce due to the oily and resinous character of hemp extracts. To solve this problem, support substances are used to form a lipid matrix for the controlled release of cannabinoids and to prevent the degradation of these compounds. Phospholipids are the most effective emulsifiers for cannabinoid-containing emulsions (mainly Δ9-THC and CBD) [96,97,98,99]. The lipidic properties of cannabinoids are also the reason why these compounds are highly susceptible to oxidation, which compromises their storage stability. Another problem for producers is to ensure homogeneity of concentration in each portion of the product. The control of water activity in the product, as well as the adjustment of proper packaging (appropriate portions, inaccessibility to oxygen and light) are the measures that will improve the quality and durability of such products. It is also necessary to develop standardized methods for the quantification of compounds and the full characterization of cannabinoids to assess bioactivity and bioavailability. In order to determine the bioavailability of cannabinoids from food, further research needs to be conducted on the food matrices used and the oils used as carriers [100]. Examples of opportunities and limitations that may have an impact on the use of cannabinoids in food production are presented in Figure 3.

Knowledge of the changes that occur during hemp oil storage under different conditions is extremely important when considering their potential use in food processing. As confirmed by Rupasinghe et al. [101], hemp seed oil is highly susceptible to rancidity caused by heat and long-term storage. Hemp seed oil contains acidic cannabinoids, especially cannabidiolic acid, which are present at the highest concentrations in oil. The ratio of CBDA to CBD present in hemp oil is a good indicator of correct oil storage conditions and production processes as well as the freshness of a particular product. Long storage times and incorrect storage conditions may lead to the degradation of CBDA to CBD [102,103].

Processing raw materials with increased temperatures, such as by drying, heating, and combustion, changes the content of cannabinoids. As a result of these processes, non-psychoactive carboxylic acids are transformed into neutral cannabinoids by decarboxylation [103,104,105]. Δ9-THC, derived from Δ9-THCA as a result of decarboxylation, is transformed into CBN under the influence of light and oxygen during further oxidation processes [96]. No enzymes participate in the decarboxylation of cannabinoid acids, which only occurs under the influence of temperature; the higher the temperature, the faster the process [17]. Methods of analysis of compounds present in a product may cause structural changes in cannabinoids, but the optimum times and temperatures for decarboxylation have not yet been determined.

In food processing, hemp seeds are mainly pressed to obtain oil and used for the production of hemp flour. They are also ground to serve as a source of plant-based protein and dietary fiber. Originally, the ground seeds and flour were added to energy bars, flavored yoghurt, and baked foods [101,106,107]. Steinbach [108] developed a production process for pralines and chocolates containing hemp seeds. In 2009, Shim [109] used hemp seed oil and hemp seeds to produce bread and confectionery products. Guang and Wenwei [110] developed a patent for hemp flour in the production of functional foods because its consumption increased the level of high-density lipoproteins and balanced the levels of other glycerides. Berghofer et al. [111] obtained hemp seed milk, which did not change color or become bitter after pasteurization. According to Bisterfeld von Merr [112], adding hemp juice to alcoholic beverages may benefit digestion. The health effects of consuming products containing hemp oil and seeds have not yet been fully established. The benefits reported so far include reductions in total cholesterol and blood pressure in those who consume these products [20]. A very important aspect concerning the use of cannabinoids is to enable the easiest possible application of these compounds to food. Considering the differences in content and quality of individual cannabinoids in plants from different geographical areas, it is necessary to apply alternative methods for synthesis and isolation of these compounds. One of these methods is the use of synthetic biology for the synthesis of cannabinoids in heterologous hosts. Due to the increasing susceptibility of plants to climate change and disease, plants may synthesize fewer cannabinoids than originally. Chemical synthesis is required to ensure the availability of these compounds. The biosynthesis of cannabinoids by modified and carefully selected microbial strains may provide an alternative to traditional methods of cannabinoid extraction. The biosynthesis of major cannabinoids requires the discovery and characterization of all key enzymes involved in the synthesis of these compounds. Synthetic biology may enable the extraction of more compounds presents in *Cannabis sativa* L. at low concentrations [113].

Nowadays, more and more consumers are interested in foods, that contain cannabinoids. As a result, producers are competing with each other to use cannabinoids in the widest possible range of products. Many aspects influence the possibilities and limitations for the use of cannabinoids in food production in the future. It is important to understand the effects of cannabinoids on the human body and its tissues. It is also necessary to determine the long-term exposure to these compounds during the consumption of cannabis foods and the possible side effects of this. Monitoring the amount and periodicity of consumption and the quality of hemp products will allow us to determine the exposure to cannabinoids. Social anxiety, prejudice, and insufficient knowledge are also barriers to the development of this type of food. It is also necessary to regulate the permissible limits of not only THC but other cannabinoids in products. Given the number of positive reports on cannabinoids and their effects on the human body, the concerns about adding them to food are unfounded. It is important to precisely characterize the properties of each cannabinoid, as the properties known so far suggest that these compounds could replace many common drugs in the future.

## 5. Analytical Techniques for Food Cannabinoids

From the perspective of food safety, the analysis of cannabinoids in food products made from hemp is extremely important. The aim of such analysis is to minimize the risk resulting from the presence of psychoactive substances that have an adverse effect on the human body. The choice of the technique for determining the presence of cannabinoids depends on the nature of certain compounds as well as the levels of these analytes in the tested material [39].

Gas chromatography coupled with mass spectrometry (GC-MS) identifies only neutral cannabinoids with low molecular weights. This leads to the decarboxylation of the acidic cannabinoids and the transformation into their neutral forms under the influence of high temperatures in a chromatography column furnace [96,114]. Derivatization is used to prevent the decarboxylation of cannabinoids. This consists of transforming molecules into a more stable and volatile compound [14,115]. Despite the required additional stage of sample preparation, GC-MS is often used because of its simplicity, speed, and high sensitivity for determining total cannabinoid content (neutral and acidic) [34,75,116]. Liquid chromatography coupled with mass spectrometry (LC-MS) allows for the identification and determination of both neutral and acidic cannabinoids without decarboxylation and derivatization, which is an advantage over GC-MS [96,117]. The determination of cannabinoids by high-performance liquid chromatography (HPLC) coupled with UV detection allows for high detection limits. It further allows for the quantification of the main compounds present in the matrix at relatively high concentrations. However, it has the disadvantages of low sensitivity and specificity, which limits the detection of cannabinoids present at low concentrations that are too high [118]. An ultraviolet (UV) detection is most frequently used for analyzing the high concentrations of cannabinoids present in plant materials [119,120]. The use of HPLC coupled to a flame ionization detector (FID) is limited as acidic cannabinoids (i.e., CBDA and Δ9-THCA) cannot be determined by this method [121]. However, mass spectrometry, particularly tandem mass spectrometry (MS/MS), is considerably more sensitive than UV detection or FID [39,102,121].

Marchetti et al. [122] used the method 13C-qNMR Spectroscopy to determine the non-psychoactive cannabinoids in fiber-type *Cannabis sativa* L. (hemp). This method provided reliable results compared to a more consolidated HPLC technique. 13C-qNMR Spectroscopy offered sufficiently precise and sensitive results, with LOQ values lower than 750 μg/ mL. This technique is suitable and advantageous for the analysis of non-psychoactive cannabinoids in hemp extracts. Moreover, it is a good alternative to the chromatographic methods and could be applied in both the plant material and its derivatives.

In 1975, Smith was one of the first to use HPLC-UV to analyze the cannabinoids present in *Cannabis sativa* L. His analyzes led to the identification of eight major cannabinoids (CBD, CBDA, CBN, Δ9-THC, CBC, cannabinolic acid, Δ9-THCA, and CBCA) [123]. In their research on the cannabinoids present in plant material, De Backer et al. [120] used HPLC with a diode array detector (DAD). This allowed for assays of the content of certain cannabinoids in plant samples, which made it possible to specify the level of psychoactive effect of a given plant and also to identify multiple types of fibrous hemp. Currently, most cannabinoid determination methods specifically relate to Δ9-THC, Δ9-THCA, CBD, CBDA, and CBN. This is related to the EU’s recommendation to regulate the content of certain cannabinoids in food [89]. The most common method of food analysis is liquid chromatography coupled with mass spectrometry because it enables assays for the content of acidic cannabinoids, which may be converted into their psychoactive neutral forms, such as through thermal processing [118].

In the literature, there is little information on the cannabinoid content of the cannabis contained in foods and beverages. Most authors concentrate on analyzing hemp itself: its inflorescences, leaves, seeds, and extracts, as well as hemp oils. RP-HPLC-UV was used for the quantitative and qualitative determination of the cannabinoids present in the two inflorescence samples of the same hemp variety, *Cannabis sativa* L. var. *sativa*. The largest differences in the content of cannabinoids were observed for CBDA (sample 1–0.88 g/100 g of inflorescence; sample 2–0.93 g/100 g of inflorescence). The contents of Δ-9-THC and Δ-8-THC were below 0.1 g/100 g of inflorescence. Δ9-THCV was not present in any sample. CBGA, which is a precursor for the synthesis of other cannabinoids, has been detected at very low concentrations. The remaining analyzed cannabinoids had similar or the same concentrations in the two samples [124]. Similar studies were carried out by Žampachová et al. [125] who analyzed the content of cannabinoids in *Cannabis sativa* L. var. *sativa* inflorescences using nano-LC-UV. As expected, the highest levels were reported for CBDA (23.8–40.9 mg/g) and CBD (5.9–32.5 mg/g). The presence of CBGA (37.5 and 31.8 mg/g) and CBG (10.9 and 2.3 mg/g) was quantified in two analyzed samples. None of the analyzed fibrous hemp samples had a Δ9-THC content exceeding 0.2% of the dry plant mass. The authors concluded that their method was faster and more selective than other methods, such as HPLC and UHPLC. Other authors reported no differences in cannabinoid content between the upper and lower parts of the inflorescence. Both Δ9-THC and CBD were present at the highest concentrations in bracts compared to the whole inflorescences. The authors also emphasized that the differences in the chemical composition between plants of the same variety depend on the growth cycle, harvest period, and the storage of harvested materials [126]. Cardenia et al. [127] developed a rapid GC/MS method for the quantification of cannabinoids present in *Cannabis sativa* L. inflorescences. The compounds with the highest concentrations were CBDA (5.2 g/100 g of inflorescence) and CBD (1.56 g/100 g of inflorescence). CBGA and CBC occurred in amounts lower than 1 g/100 g of inflorescence. The remaining cannabinoids were either not detected or detected at very low concentrations. Jang et al. [128] analyzed commercially available hemp seeds and hemp seed oil for Δ9-THC, CBD, and CBN content. In hemp seeds, the concentration of Δ9-THC ranged from 0.06 to 5.91 mg/kg of seeds, the CBD concentration was within 0.32–25.55 mg/kg, while CBN ranged from 0.01 to 1.5 mg/kg. In commercially available hemp oils, the determined Δ9-THC content ranged from 0.3 to 19.73 mg/L, CBD from 6.66 to 63.40 mg/L, and CBN from 0.11 to 2.31 mg/L. The low CBN concentration in all the analyzed samples indicates the freshness of the seeds and oil as well as the storage conditions.

To ensure the safety of food and finished food products containing cannabinoids, it is necessary to quantify the cannabinoids both as a raw material and as the finished product. A comparison of the methods used for these analyses is shown in Table 4.

Lachenmeier et al. [92] used LC-MS/MS to determine the content of ∆9-THC in 67 food samples. As much as 25% contained ∆9-THC at concentrations exceeding the lowest observed level of adverse effect, which was 2.5 mg ∆9-THC/day, while 43% were classified as unsuitable for consumption because they exceeded the ARfD of 1 µg ∆9-THC/kg [85]. Christinat et al. [39] used LC-MS/MS to analyze the presence of 15 cannabinoids in hemp oil and seed samples, as well as in tea, coffee, chocolate, mayonnaise samples, and one milk sample collected from cows fed with hemp. The study demonstrated that products belonging to the same category may have very different cannabinoid profiles and levels, although components from a *Cannabis* plant were added to each product, accounting for 10% of the entire product composition. The CBD content in cow’s milk was 0.0095 mg/kg, and the compound was never determined in this matrix. In mayonnaise, none of the cannabinoids were measured above the limit of quantification. Teas, coffees, and chocolates containing hemp leaves or flowers were characterized by a higher content of cannabinoids than products containing hemp seeds. This is not surprising because leaves and inflorescences contain much more cannabinoids than hemp seeds. Brighenti et al. [133] developed a method for quantifying the non-psychoactive cannabinoids (CBDA, CBGA, CBG, and CBD) in *Cannabis sativa* L. inflorescences and hemp oils, as well as in ethanolic hemp-based extracts and hemp balms, using HPLC-UV/DAD (diode array detector). The CBDA and CBD content of hemp inflorescences ranged from 0.1 to 46.8 mg/g and from 0.1 to 23.9 mg/g of dry matter, respectively. CBGA is a precursor for the synthesis of other compounds; therefore, its content in inflorescences amounted to <9.5 mg/g dry matter, while CBG was <6.5 mg/g dry matter. In the hemp oils, the highest concentration (78.6 mg/mL) was reported for CBD. The remaining cannabinoids were not detected in the oils, or they were present in small amounts. Only CBD (193.7 mg/g) was present in the ethanolic hemp extract. With hemp balm, the content of CBDA amounted to 44.7–80.4 mg/g of the product, CBD amounted to 7.6–13.8 mg/g, while CBGA and CBG were present in considerably lower concentrations, 2.0–3.9 mg/g and 0.4 mg/g, respectively. One of the hemp balms did not contain CBGs. Ciolino et al. [132], by using HPLC-DAD, also quantified 11 cannabinoids in 60 food products, mainly dietary supplements and beverages. This method can be used to quantify these compounds in multiple food products (e.g., candies, beverages, oils, and dietary supplements) as well as in extracts and plant formulations. In commercial buds, the dominant cannabinoid was Δ9-THCA (138–241 mg/g). Among all the dietary supplement samples analyzed by the authors, the most abundant cannabinoid was CBD (144–350 mg/g). However, the precursor of CBD (CBDA) was not detected or was present in small amounts, which may indicate that the production process involved a thermal processing that led to the decarboxylation of CBDA. In other studies by the same authors, GC-MS was used to quantify 11 cannabinoids present in the extract samples from different parts of the plant as well as in food (candies, beverages, dietary supplements, and hemp oils). In extracts of fresh hemp inflorescences, the content of Δ9-THC was 10% lower than the content of Δ9-THCA. A considerably higher Δ9-THC content was observed in hemp oils. The dominant cannabinoids in hemp seed oils were CBDA and CBD, while Δ9-THC and Δ9-THCA were present in small amounts. CBN was not detected in the oil samples. The CBD content in dietary supplements was 145 mg/g. Dietary supplements also contained small amounts of other cannabinoids (CBDV, Δ9-THC, CBC, CBG, CBDA, and CBN), up to 5 mg/g of the product. Detecting cannabinoids that are present in lower concentrations than CBD can provide valuable information on the quality of the added hemp extracts (degree of enrichment with a certain ingredient and degree of decarboxylation of acidic cannabinoids). After analyzing the ethanol extracts up to 3 days after their preparation, it was found that all the cannabinoids were stable [75]. Pisciottano et al. [105] developed a method for the determination of Δ9-THC, Δ9-THCA-A, Δ8-THC, CBN, CBD, CBDA, CBG, CBGA, and Δ9-THCV in food, beverages, and animal fodder. Their analysis included 100 samples of products from the Italian market, including 78 food products and 16 beverages. CBD and CBDA were the most frequently identified cannabinoids (78% and 84%, respectively). Samples of hemp seed flour and the flour mixture had high concentrations of CBD, ranging from 1.48 to 14.7 mg/kg. A high concentration of Δ9-THC and Δ9-THCA aggregates was determined in hemp seeds and flour (2.0 mg/kg), oil (5.0 mg/kg), and dietary supplements (2.0 mg/kg). The acidic form of CBDA was found more frequently in beverages. A significant amount of Δ9-THCV and CBN (> 1 mg/kg) was found in honey samples with added CBD and a herbal mixture containing hemp. In their studies, Citti et al. [102] demonstrated that hemp oils are characterized by a total Δ9-THC content ranging from 5 to 10 mg/kg. This value is higher than the legal limits in many countries only if the aggregate content of Δ9-THCA and Δ9-THC are taken into account. If only neutral Δ9-THC is analyzed, then its content in the samples generally does not exceed 2 mg/kg. Escrivá et al. [134] analyzed the occurrence of Δ9-THC in milk and infant formula as well as in hemp seeds. In three out of five samples of infant formula, the Δ9-THC content ranged from 4.76 to 56.11 ng/g. In the samples of hemp seeds that were used as an ingredient of cow fodder, 0.82 mg Δ9-THC/kg of seeds was determined. No Δ9-THC was detected in the milk samples. Lee et al. [135] used LC-MS/MS to quantify CBD and Δ9-THC content in products containing cannabinoids divided into three groups: group 1 (high fat)—chocolate, energy bars, and oils; group 2 (high sugar)—candies and jellies; and group 3 (other products)—powdered hemp protein, snacks, and cereals. The QuEChERS (Quick, Easy, Cheap, Effective, Rugged, and Safe) method was used to clean the samples, which is often used for extracting samples to the solid phase or to clean them to eliminate matrix contamination (e.g., sugars, organic acids, lipids, and fatty acids). Of the 30 food samples containing cannabinoids, CBD was detected in 15 samples in amounts ranging from 70 to 31.31 mg/kg. The Δ9-THC content in 12 food products was 0.08–98.62 mg/kg. Both chocolate and hemp oil samples featured the highest CBD and Δ9-THC content among the analyzed products. In their research, Fernández et al. [130] compared the contents of CBD, CBN, and Δ9-THC in hemp oils with a declared CBD content of 20 mg/mL (two samples) and in oils without any of the above, defined cannabinoids (eight samples), using GC-MS. In both oils, the CBD concentrations (22 mg/mL) were consistent with those declared by the manufacturer. The Δ9-THC and CBN concentrations in these oils were very low (1 mg/mL). The results for the remaining oils differed significantly from each other. Only three oil samples from this group contained CBD, with concentrations ranging from 0.3 to 1.5 mg/mL. A considerably higher discrepancy was observed for Δ9-THC (0–29 mg/mL) and CBN (0–3.4 mg/mL). Such a high Δ9-THC content can, with excessive consumption of a given oil, induce narcotic effects (especially in children) [137]. Pellegrini et al. [131] used GC-MS to quantify CBD, Δ9-THC, and CBN in selected food samples containing cannabinoids. The highest Δ9-THC content was observed in scented grass (350 ng/g) and hemp seeds (328 ng/g). CBD was not detected in the seeds, whereas a high concentration was detected in the liqueur. Beer and liqueur did not contain CBN, while scented grass had the highest concentration (160 ng/g). Romano et al. [136] conducted two independent experiments involving mead fermentation with the addition of each plant component separately (inflorescences, leaves, and stems) as well as with the addition of their blends in different proportions of individual plant components at 0.25 and 0.50% (*w/v*). The meads were fermented for five weeks at 25 °C. After fermentation, HPLC-FID analysis was conducted to quantify CBD and CBN in the finished products. CBD was detected at 0.26 mg/L and 0.49 mg/L. This compound naturally occurs in the greatest amount in inflorescences. No CBN was detected; this is mainly produced by Δ9-THC degradation, while the plant material used in the experiments contained less than 0.2% of Δ9-THC in dry matter. Dubrow et al. [122] analyzed the content of selected cannabinoids using HPLC-DAD in random products from the US market. The authors analyzed 147 products that were divided into the following groups: tinctures and oils, powders and capsules, food products (honey, candies, and cookies), jellies, beverages, and products for animals. CBD accounted for >98% of the cannabinoid profile in 46 samples, which suggests that the products were made using CBD isolates or highly purified extracts, such as jellies (14.05 mg/g). The highest Δ9-THC concentration among the analyzed samples was reported for the tinctures and oils group (3.34 mg/g); in most samples, Δ9-THCA was not detected or it did not exceed 0.1 mg/g of the product. CBN was present in the tinctures and oils group (1.42 mg/g). Pavlovic et al. [45] assessed the quality of 14 oils containing CBD, commercially available in European countries. Nine of the samples had CBD concentrations higher than their declared content. The CBD content in the oil samples fell within 1875 to 48,879 mg/kg, indicating an enormous variability and suggesting that stricter regulations relating to CBD content in food products need to be implemented. The authors also quantified Δ9-THC and CBN. The Δ9-THC content exceeded 0.2% in only one of the analyzed samples, although the manufacturer declared that the product did not contain Δ9-THC. Most samples also contained CBN, a product of Δ9-THC oxidation. The detection of this compound in hemp oil suggests that it was produced through an improper process or extraction processes, or that the finished product was improperly stored.

The analysis of the cannabinoids present in food entails challenges related to the matrices in which these compounds are present, as well as the correct choice of how samples must be prepared for the chosen technique. As cannabinoid content differs by 25% within a single cannabis strain, the use of these plants in food production necessarily generates differences in the cannabinoid profile in finished products [9]. There is a clear need to develop effective and efficient cannabinoid assays. GC-MS is the most frequently used technique to determine cannabinoids; however, the quantitative determination of these compounds requires an additional derivatization process to prevent the decarboxylation of acidic cannabinoids [14,34,138]. HPLC-DAD and HPLC-UV constitute good alternatives to GC, but the specificity and sensitivity of these techniques are much lower than those of GC. Researchers often analyze cannabinoids using LC-MS/MS, which can be used to determine both acidic and neutral compounds without affecting their structure and degradation degree [78,138].

## 6. Cannabinoid Stability

Current data relating to the stability of individual cannabinoids are limited. Most authors have concentrated on two main compounds, Δ9-THC and CBD. There is still a lack of knowledge on the products formed by the thermal degradation of cannabinoids; these require characterization and toxicological assessments. To ensure the safety of cannabinoid-containing products, the cultivation conditions of the plants from which the extracts are obtained should be standardized in the region and the stability of the extract during storage should be checked [92]. As confirmed by Lindholst [139], from a chemical point of view, using ethanol or methanol as solvents for cannabinoids improves their stability, while the opposite effect is achieved with chloroform. Extracts derived from seeds, resin, or inflorescences containing cannabinoids can be temporarily stored in chloroform. Understanding how cannabinoids change during thermal treatment or storage under improper conditions is extremely important because cannabinoid degradation products can potentially demonstrate health benefits or have an adverse effect on human health. In the future, adding these compounds to food will mean fulfilling all labeling requirements for cannabinoid-containing products [140].

### 6.1. Cannabinoid Stability with Respect to Temperature, Time, and Light

The first reports relating to the effect of temperature and light on cannabinoid stability were presented by Fairbarin et al. in 1976 [141]. These studies involved the storage of dried hemp leaves and dried hemp inflorescences ground together at 5 °C without light and at 20 °C with and without light for 98 weeks. The analyses also included samples of powdered cannabis resin stored in packaging at 20 °C with and without light. During storage at 5 °C in the dark, the Δ9-THC content in the samples of ground leaves and inflorescences decreased by 10%. Storing the leaf and inflorescence samples at 20 °C in the dark caused the Δ9-THC content to decrease by 25%, while for the samples stored in light, the content dropped by 63%. Powdered cannabis resin was collected from the surface, from a layer 2 mm below the surface, and from the middle of the package in which the resin was stored. The Δ9-THC content on the powder surface decreased from 11.6 to 5.2%. However, in samples collected 2 mm below the surface and from the middle, the content decreased to 11.4% [141]. Grafström et al. [142] emphasized the role of oxygen in the cannabinoid degradation in resin. They observed that the resin samples stored with access to air exhibited much faster degradation of Δ9-THC and CBD, both in the presence and absence of light. They found that CBD was more stable than Δ9-THC regardless of the storage conditions (light and temperature). Trofin et al. [143] showed that both temperature and light affect the stability of cannabinoids (Δ9-THC, CBN, and CBD) extracted from resin samples using methanol:chloroform (9:1, *v/v*) extraction. In the first year of storage at 22 °C with light (variant 1), the degradation of Δ9-THC was 1.02 times higher than that of samples stored in the dark under refrigerated conditions (4 °C) (variant 2). CBN formation in the same samples stored according to variant 1 was 1.10 times faster than that of samples stored according to variant 2. An opposite relationship was observed for CBD content. With variant 1 storage, the CBD concentration of this compound was reduced by 0.62 times compared to the samples stored in the dark at 4 °C. Lindholst [139] assessed the stability of four cannabinoids (CBN, CBG, CBD, and Δ9-THC) during the storage of resin and resin extracts (20–22 °C with and without light and −20 °C without light). Cannabinoids were also extracted using methanol:chloroform at a ratio of 9:1 (*v/v*). The initial cannabinoid concentrations in the resin were 0.4, 0.4, 3.5, and 11.7%. During the storage of resin samples and resin extracts for 4 years, the total Δ9-THC concentration decreased in samples exposed to light (up to 1–2%). In resin samples stored in the dark for the first 240 days, there was an increase in the Δ9-THC concentration, which, in subsequent years of storage, was only slightly lower than the initial concentration. The extract samples were characterized by higher Δ9-THC degradation than observed in the resin samples stored in daylight. In all the resin samples, CBD and CBG remained stable for the entire period of storage in the dark at −20 °C. An increase in CBN along with a decrease in total Δ9-THC was observed during storage at 20 °C, but no direct correlation was observed between CBN synthesis and Δ9-THC degradation. Lindholst [139] also carried out studies related to the storage of hemp resin extracts. The half-life of acidic Δ9-THC in the resin extracts was 10 times shorter than that observed in hemp resin samples. This could be due to differences in sample volumes, which could lead to increased light exposure, contributing to faster degradation. No significant differences were observed for hemp resin extracts during dark storage. The changes in Δ9-THC concentrations may be a consequence of the degradation into other compounds in addition to CBN.

In another study, Trofin et al. [144] analyzed the effect of storage conditions on the concentrations of Δ9-THC, CBD, and CBN in hemp oil samples. Cannabinoids were extracted using methanol:chloroform (9:1, *v/v*). The hemp oil samples were stored for 4 years at 4 °C with and without light at 22 °C in dark glass bottles. The Δ9-THC concentration decreased in all samples during storage. A higher reduction in the Δ9-THC content was observed when the samples were stored in light at 22 °C (content reduction of 89.9%) compared to refrigerated conditions (4 °C) in the dark (content reduction of 83.8%). In addition, an increase in the CBN concentration was observed regardless of the storage conditions. In samples stored in light at 22 °C, the concentration increased by 87.2%. Under refrigerated conditions, the concentration of this compound was reduced by 83.2%.

Citti et al. [102] analyzed the effect of temperature and storage time on the stability and decarboxylation degree of CBDA in hemp oil. The samples were stored at 5, 20, and 25 °C. The half-life of CBDA in the hemp oil samples stored at 5 °C was approximately 20 months. When the oil was stored at 20 and 25 °C, the half-lives were 49 and 20 days, respectively. Meija et al. [145] conducted stability testing on seven cannabinoid pairs (CBC, Δ9-THC, CBN, CBG, CBD, Δ9-THCV, and CBDV, as well as their acidic forms) in dried hemp material stored in the dark at temperatures ranging from −20 to 40 °C). The average monthly degradation of Δ9-THCA + Δ9-THC was 2% at 20 °C. It was observed that the storage of these compounds at 4 °C did not ensure long-term (more than 12 months) cannabinoid stability. Zamengo et al. [146] defined the average degradation of Δ9-THC within 100 days, which amounted to 12% at 22 °C (3–4% per month).

Peschel [147] examined the stability of CBDA, CBD, Δ9-THC, Δ9-THCA, CBG, and CBGA in two hemp variants during the storage of hemp tinctures (20 °C with light and 4 °C without). Storage with exposure to light at 20 °C contributed to the increased decarboxylation of Δ9-THCA, CBDA, and CBGA compared to storage in the dark at 4 °C. The author also showed that neutral cannabinoids degraded slower at 4 °C than at a higher temperature. The main process that occurs during the long-term storage of hemp tinctures is the decarboxylation reaction of cannabinoids. After storing the tinctures for 3 months at 20 °C with exposure to light, both Δ9-THCA and Δ9-THC were completely degraded. During 15 month storage at 4 °C, a decrease in the content of Δ9-THCA was observed while a simultaneous increase in the content of Δ9-THC was observed, but the aggregate content of these compounds decreased. The aggregate content of CBGA and CBG during both storage variants did not change significantly.

Pacifici et al. [148] analyzed the stability of cannabinoids present in hemp tea and hemp oils. During the storage of tea at 20 °C, a significant reduction (<50%) was observed as compared to the initial Δ9-THC and CBD concentrations after 3 and 7 days, respectively. The stability of Δ9-THCA, CBDA, and CBN was observed up to 14 days. At 4 °C, the reduction in the content of Δ9-THC, CBN, CBG, and CBC amounted to 65% after 3 days of storage. CBDA remained stable for 14 days. For Δ9-THCA and CBD, their concentrations decreased by 70 and 40%, respectively, after 7 days of storage. For hemp oils, the highest drop (28%) in Δ9-THC content was observed for oil made from inflorescences that had not been previously heated after 14 days of storage at 20 °C. For all the remaining cannabinoids, losses from 80 to 85% were reported after 14 days of storage, regardless of the storage conditions. Under all storage conditions, hemp oil showed the highest stability.

Milay et al. [149] attempted to determine the optimum storage conditions (1 year) for dried hemp inflorescences and their extracts to maintain cannabinoid content. The samples were stored in the dark at different temperatures (−80, −30, 4, and 25 °C), whereas for extracts, the solvents also differed (olive oil, ethanol, or dimethyl sulfoxide). For both inflorescences and extracts, storage at 25 °C caused the greatest changes in cannabinoid content, which made such conditions the most unfavorable for storing samples containing these compounds. The storage of whole inflorescences and extracts at 4 °C is optimal for maintaining the natural cannabinoid content in the samples. The use of olive oil as a solvent improves cannabinoid stability. The acidic cannabinoids present in inflorescences were much less susceptible to decarboxylation than hemp extracts under the same storage conditions, depending on the solvent used. In ground inflorescence samples, the decarboxylation process was more intense than in the whole inflorescence. McRae and Melanson [119] stored samples of extracts obtained from dried and ground inflorescences at −20 °C for 8 weeks, demonstrating that these storage conditions maintain the stability of the cannabinoids extracts over this period. Despite the use of very low temperatures and a limited exposure to light, the authors observed slow decarboxylation after 8 weeks.

Mudge et al. [150] assessed the stability of cannabinoid standards dissolved in 80% methanol and stored at −20, 4, and 22 °C without light access. The standards were prepared separately and mixed. For separately prepared standards, significant changes in the content of Δ9-THCA and CBDA were observed after 30 h at 20 °C, and amounted to 6.3 and 9.6%, respectively. The standards mixed and stored at −20 °C were degraded after 48 h of storage and the reduction in the contents of Δ9-THCA and CBDA content amounted to 8.1 and 10.6%, respectively. Additionally, the research compared the stability of inflorescence extracts prepared using 80% methanol and a mixture of methanol and chloroform (9:1, *v/v*) during storage in the dark at 4 and 22 °C. It was concluded that methanol:chloroform extracts were stable at 4 °C for 48 h and 22 °C for 36 h. Extracts in 80% methanol were also stable at 4 °C for 48 h, but increasing the temperature led to reduced cannabinoid stability after 24 h.

Based on the above results, it can be concluded that the longest stability of cannabinoids, both neutral and acidic, requires them to be stored in the dark. The use of refrigerated temperatures reduces the loss of cannabinoids during storage caused by decarboxylation.

### 6.2. Cannabinoid Stability with Heating

The available knowledge regarding the stability of cannabinoids during heating is insufficient to confirm that products containing cannabinoids are a safe additive or ingredient. There is a lack of data on the stability of these compounds during processing, and the degradation products of these compounds are not fully known. One of the studies carried out by Turek and Florian [151] confirmed that each increase in heating temperature of hemp oil causes a twofold increase in the cannabinoid degradation rate. Thermal degradation and oxidation are reactions that lead to undesirable changes in edible oils, including hemp oil. The knowledge of the effect of both reactions on the quality of hemp oil is essential to maintain the proper quality of oils used for food production. According to many studies, hemp oil is considered unstable because it contains large amounts of unsaturated acids (e.g., α-linoleic acid and γ-linolenic acid), which are susceptible to oxidation during heating and storage [152,153,154].

Dussy et al. [115] heated pure Δ9-THCA for 15 min at 160 °C, which resulted in the complete conversion of Δ9-THCA to Δ9-THC, while CBN was additionally formed during heating at 180 °C. The decarboxylation rate constant determined by Wang et al. (2016) for Δ9-THCA was twice as high as those for CBDA. The conditions under which the synthesis Δ9-THC reached its maximum efficiency were: 10 min at 145 °C, 15 min at 130 °C, and 20 min at 110 °C. In contrast, the maximum concentration for CBD was achieved after heated under the following conditions: 10 min at 145 °C, 30 min at 130 °C, and 50 min at 110 °C.

Citti et al. [102] examined the decarboxylation of compounds present in hemp oil when heated. The experiment was carried out in two variants: open and closed vials with hemp oil. The first variant involved heating the samples in an oven; after 15 min, one vial was removed, cooled down to room temperature, diluted, and analyzed using HPLC-UV. This was repeated at 80, 90, 100, 110, and 120 °C. The second variant involved heating closed vials containing hemp oil in an oven at 120 °C; after 15 min, one vial was removed, cooled down to room temperature, diluted, and analyzed using HPLC-UV. The results obtained for the first variant showed that at temperatures below 100 °C, the aggregate concentrations of CBD and CBDA were not subject to significant changes (a drop of 1% and 2%). The decarboxylation of CBDA below 100 °C led to the formation of CBD. Using a temperature of 100 °C led to a 20% decrease in the aggregate content of these compounds. Heating at 110 °C and 120 °C caused the aggregate content of these compounds to drop by 30 and 60%, respectively. At 120 °C, CBDA decarboxylation and CBD degradation were simultaneously observed. It was concluded that the higher the process temperature, the more the CBD concentration decreased, as was the aggregate content of the two compounds formed. During the reaction carried out in closed vials at 120 °C, a linear decrease in CBDA content was observed with the simultaneous formation of CBD, although the loss of its aggregate concentration was about 11%. These experiments demonstrated that both the increased temperature and oxygenation during heating affect CBDA decarboxylation, and the influence of these factors also leads to the degradation of neutral CBD.

Casiraghi et al. [155] analyzed samples of hemp inflorescences subjected to high temperatures (85–145 °C) before cannabinoid extraction. They concluded that the optimal temperature was 115 °C. Under such heating conditions, the complete decarboxylation of Δ9-THCA to Δ9-THC occurred within 40 min and did not lead to the formation of CBN. According to the authors, heating the inflorescences before further extraction makes it possible to obtain extracts or oils with high concentrations of neutral cannabinoids that affect the human body, unlike the acidic cannabinoids.

Pacifici et al. [148] analyzed the content of CBDA and Δ9-THCA in hemp oil obtained from hemp inflorescences pre-heated in an oven at 145 °C for 30 min. Pre-heating led to the complete decarboxylation of CBDA and Δ9-THCA, but no significant increase in Δ9-THC concentration was observed in contrast to CBD, which resulted from the degradation of CBDA.

Taschwer and Schmid [156] carried out stability tests on Δ9-THCA and Δ9-THC at different temperatures (50, 100, and 150 °C) for samples of dried and ground hemp inflorescences. The samples (stored at 50 °C) were collected every 2 h for the first 8 h; in the other temperature variants (100 and 150 °C), the samples were collected every 1 h for the first 8 h. The final samples were collected 24 h after the start of the storage period. The contents of individual cannabinoids in the sample were expressed as percentages. A slight effect of the temperature of 50 °C on the decarboxylation process was observed (Δ9-THCA concentration changed from 12.21 to 11.69%). The same conditions for Δ9-THC led to an increase in concentration from 1.51 to 2.12%, which was caused by the decarboxylation of Δ9-THCA. The conditions of 100 °C and 150 °C caused the complete decarboxylation of Δ9-THCA within 2 h and 1 h, respectively. For Δ9-THC, at 100 °C the maximum possible compound concentration was achieved, which was 12.28%, but this reduced to 4.79% after 24 h. However, as a result of heating Δ9-THC at 150 °C, the maximum possible concentration of this compound (12.77%) was achieved after only one hour, but after 24 h, the concentration had dropped to 0.19%. The research showed that the storage of hemp samples at −25 °C for 4 months did not cause changes in the content of Δ9-THCA and Δ9-THC during and after the storage period.

Peschel [147] studied the influence of pasteurization on the cannabinoid content (total cannabinoids, the aggregate content of Δ9-THC, Δ9-THCA, and CBN) in ethanol-based tinctures at 70 °C for 2 h and at 80 °C for 20 min. They found that both pasteurization methods affected the total cannabinoid content or the aggregate content of Δ9-THC, Δ9-THCA, and CBN in tinctures, resulting in a maximum loss of 20%. Pasteurization did not accelerate the Δ9-THCA decarboxylation reaction.

Knezevic et al. [157] measured residual cannabinoid (CBDA, CBD, Δ9-THCA, Δ9-THC, and CBN) in teas prepared from fibrous hemp. The samples were prepared at different temperatures (43, 55, 70, 85, and 97 °C), volumes (59, 100, 150, 200, and 241 mL), and times (6, 10, 15, 20, and 24 min). The initial CBDA content in the plant material was 4073 mg/kg. The CBDA content decreased from 1524 mg/kg at 43 °C (6 min) to 617 mg/kg at 97 °C (24 min). The concentration of CBD increased as the brewing temperature increased. The initial level was 802 mg/kg, while after brewing at 97 °C (24 min) it increased to 1710 mg/kg. The Δ9-THCA residue concentration in the infusion was only affected by the water temperature. The lowest Δ9-THCA (29 mg/kg) content was recorded after brewing at 43 °C (6 min), with an initial value of 111 mg/kg. The Δ9-THC was present in the plant material before brewing at 76 mg/kg. The concentration changed with the brewing temperature and time. It was concluded that brewing at 97 °C (24 min) ensured the highest Δ9-THC (116 mg/kg) content in tea. Brewing the hemp teas at 70 °C (15 min/150 mL) slightly increased the CBN content to 65 mg/kg compared to the initial value of 52 mg/kg.

Ryu et al. [158] compared the loss of neutral cannabinoids in hemp inflorescences with the loss of cannabinoids obtained from their extracts when heated at a maximum of 135 °C for 30 min. In all cases, the content of CBD and Δ9-THC increased, but in the case of hemp inflorescences, the content of neutral cannabinoids increased with time and did not decrease after 30 min. In the inflorescence extracts, the increase in the cannabinoid concentration was much lower than that in the fresh material, while at the end of the heating process, both CBD and Δ9-THC decreased.

Despite many studies relating to the stability of cannabinoids in hemp extracts and plants containing these compounds when processing plant material, there remain many questions to be resolved, particularly regarding the effect of oxygen when heating certain cannabinoids. Recent reports on the stability of cannabinoids in food matrices show that the environment in which we store products containing cannabinoids, the heating temperature, and matrix affect the stability of cannabinoids in the finished product. One of the recent studies looks at the stability of these compounds in chocolate. The influence of the matrix on the stability of the cannabinoid molecule is shown. The degree of matrix (chocolate) interference depends on two factors: the chemical composition of the chocolate and the chemical structure of the cannabinoids [159]. Another study determining the stability of cannabinoids in brownie found no significant effect of matrix ingredients on the stability of individual cannabinoids in the final product [160]. There is no definitive information in the literature regarding the effect of the matrix components of a given product on the stability of cannabinoids. It is extremely important to conduct such studies in order to determine the stability of these compounds in various food matrices. The methods of producing and heating of final products containing dried hemp, hemp seeds, or hemp oil, which contain cannabinoids, need to be accurately analyzed, as well as their potential degradation or transformation in the presence of other food ingredients.

## 7. Conclusions

*Cannabis sativa* L. is gaining popularity for its biological properties, which are primarily associated with both psychoactive and non-psychoactive cannabinoids. Hemp seeds and their oils are also recognized for their high nutritional and health-promoting properties. Current knowledge on *Cannabis sativa* L. var. *sativa* is very broad and allows for its accurate characterization. The constantly increasing interest in hemp and its compounds has led to human health safety concerns associated with the use of dietary supplements, dried hemp, and foods containing cannabinoids. Over the past decade, many studies on the properties of cannabinoids have opened up new possibilities for using them, other than Δ9-THC and CBD, in addressing multiple human health problems. Although our knowledge of the effects of the main cannabinoids (CBD, Δ9-THC, and CBG) on the human body is very broad, there is still insufficient information about the bioactivity of the lower concentrations of cannabinoids that are formed through the transformations of the main cannabinoids. Expanding this knowledge will allow for a wider use of this broad group of compounds in food production, potentially for functional foods. Based on the available knowledge, it is not possible to choose a clear-cut technique for quantifying cannabinoids in all matrices. Depending on the product, it is necessary to adjust the methods to the matrix in which the compounds of interest are present. There are numerous discrepancies in the quantifications of cannabinoids in inflorescences and hemp oils, likely caused by environmental conditions and the plant developmental stage. Similar correlations have been observed in studies on food. Depending on the *Cannabis sativa* L. var. *sativa* plant part, hemp oil, or extract, there are significant differences in final concentrations of cannabinoids in finished food products. Our current knowledge on cannabinoid stability during heating or storage under different conditions is not sufficient to determine the level of degradation of these compounds. There is a lack of information on the effects of food matrix ingredients and the effects of technological processes (e.g., fermentation) on cannabinoid stability. It is necessary to develop optimal methods for the processing of foods containing such compounds. Defining any strategies for handling finished products (storage time and conditions) will enable the preservation of their bioactive ingredients and thus the preservation of the benefits of this type of food. Nowadays, the most significant unknown is the cannabinoid degradation products present in food; therefore, cannabinoid degradation pathways need to be studied to ensure food safety and maintain the trust of consumers. Expanding our currently inadequate knowledge on hemp will allow for the safe use of cannabinoids in food production, including functional food, which is currently attracting much interest among consumers.

## Figures and Tables

**Table 1:** Chosen cannabinoids isolated from *Cannabis sativa* L. [1,14,30,33,34,35].

Compound Name	Acronym	Molecular Formula	Molecular Weight (g/mol)	Structural Formula	Compound Nature
Cannabigerol	CBG	C_21_H_32_O_2_	316.48	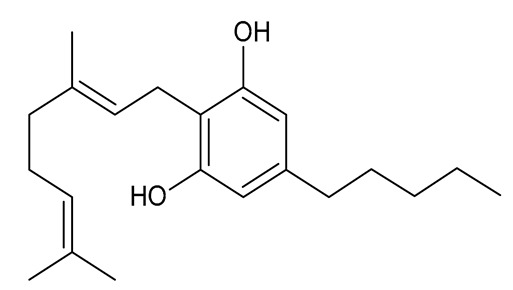	neutral
Cannabigerolic acid	CBGA	C_22_H_32_O_4_	360.49	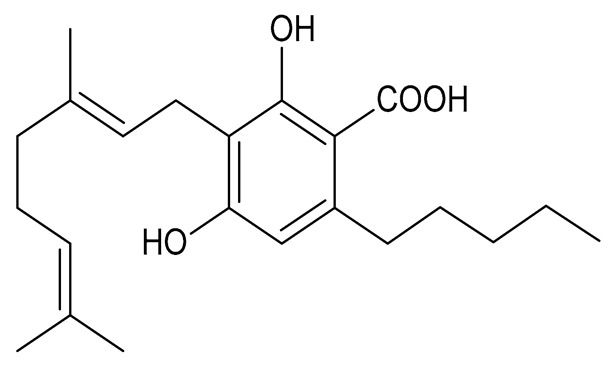	acidic
Cannabidiol	CBD	C_21_H_30_O_2_	314.46	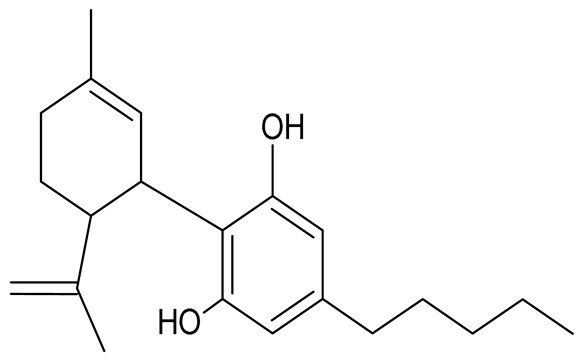	neutral
Cannabidiolic acid	CBDA	C_22_H_30_O_4_	358.47	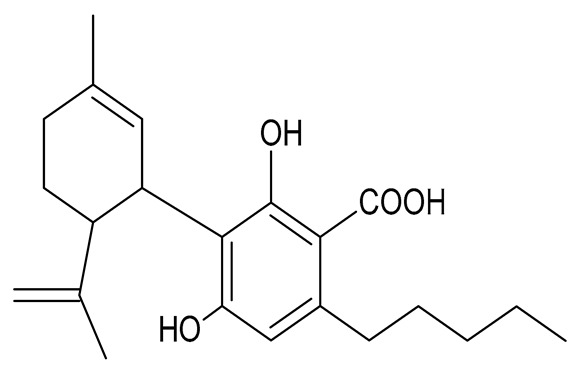	acidic
Δ9-Tetrahydrocannabinol	Δ9-THC	C_21_H_30_O_2_	314.46	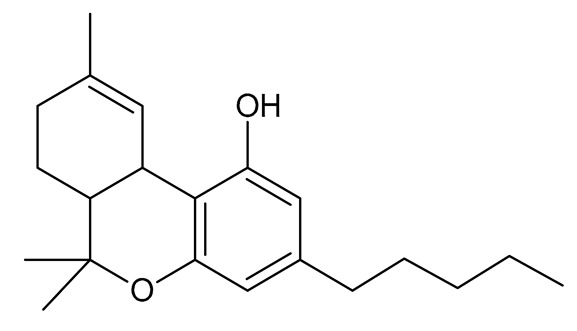	neutral
Δ9-Tetrahydrocannabinolic acid A	Δ9-THCA	C_22_ H_30_ O_4_	358.47	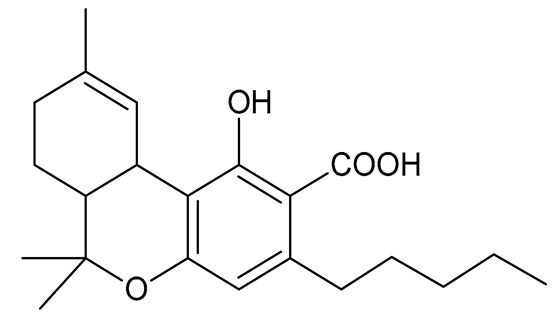	acidic
Δ8-Tetrahydrocannabinol	Δ8-THC	C_21_ H_30_ O_2_	314.46	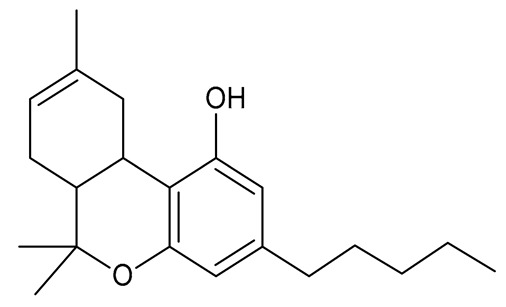	neutral
Cannabichromene	CBC	C_21_H_30_O_2_	314.46	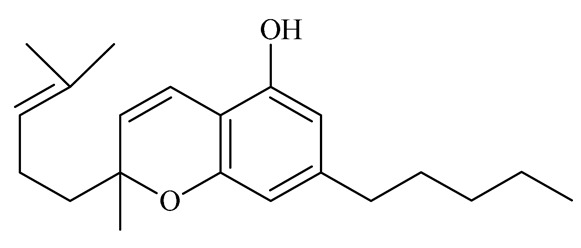	neutral
Cannabichromenic acid	CBCA	C_22_ H_30_ O_4_	358.47	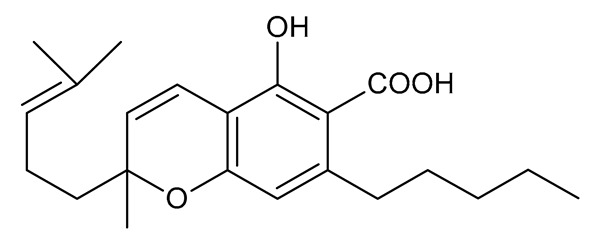	acidic
Cannabinol	CBN	C_21_ H_26_ O_2_	310.43	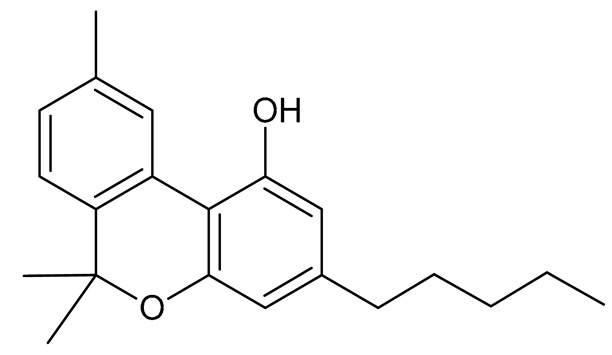	neutral
Cannabinolic acid	CBNA	C_22_ H_26_ O_4_	354.44	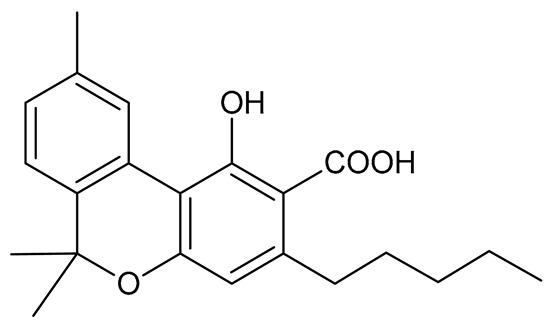	acidic
Cannabicyclol	CBL	C_21_H_30_O_2_	314.46	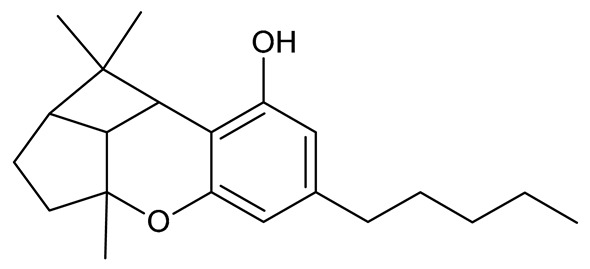	neutral
Cannabicyclolic acid	CBLA	C_22_H_30_O_4_	358.47	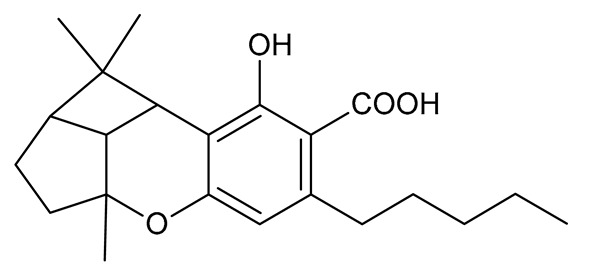	acidic
Cannabivarin	CBV	C_19_ H_22_ O_2_	282.38	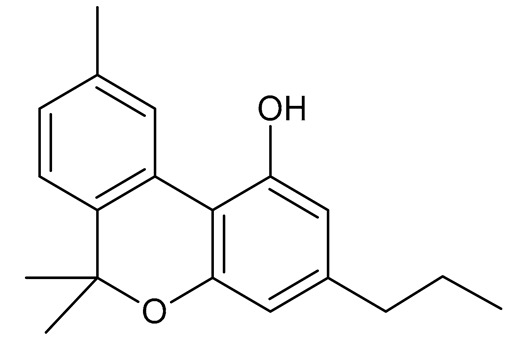	neutral
Cannabidivarin	CBDV	C_19_ H_26_ O_2_	286.41	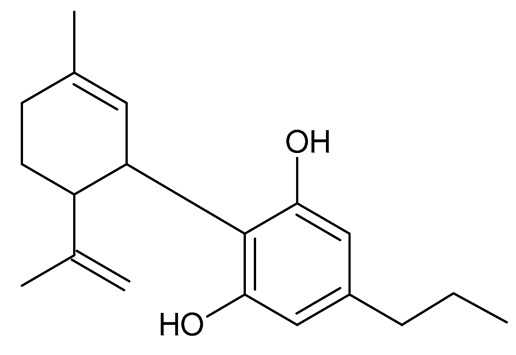	neutral
Cannabidivarinic acid	CBDVA	C_20_ H_26_ O_4_	330.42	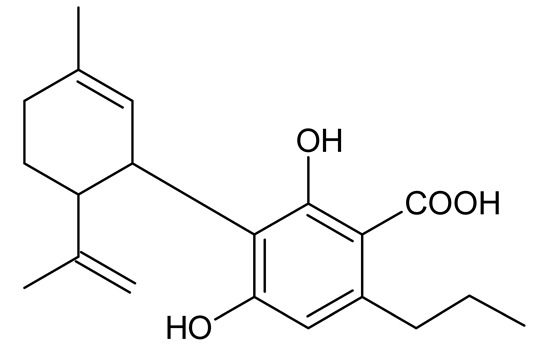	acidic
Cannabielsoin	CBE	C_21_ H_30_ O_3_	330.46	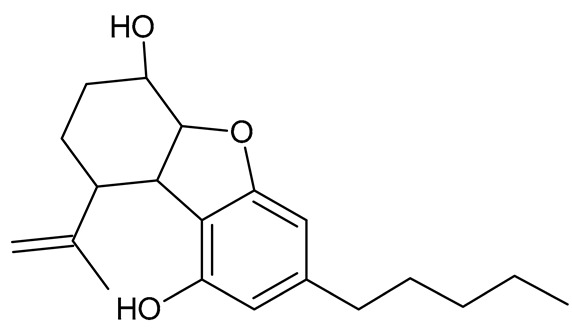	neutral
Cannabitriol	CBT	C_21_H_30_O_4_	346.46	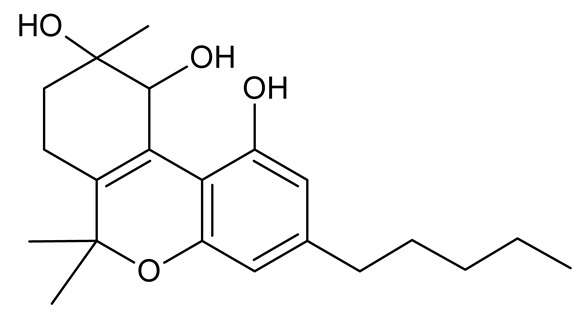	neutral
Cannabinodiol	CBDL	C_21_H_26_O_2_	310.43	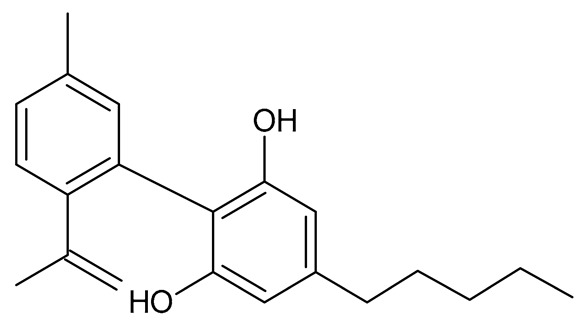	neutral
Δ9-Tetrahydrocannabivarin	Δ9-THCV	C_19_H_26_O_2_	286.41	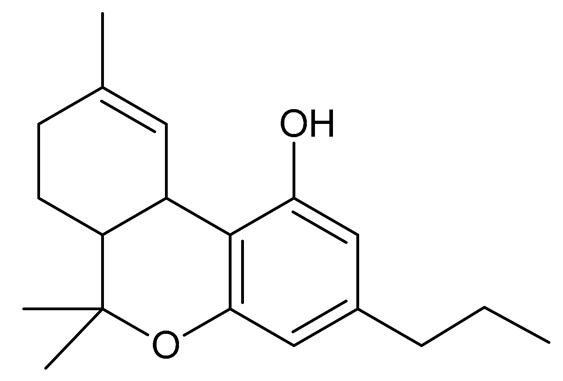	neutral

**Table 2:** Properties of selected neutral cannabinoids.

	Compound Name (Acronym)	Potential Health Benefits	Psychoactive Effects on the Human Body	*Cannabis sativa* L. Variety in Which the Compound Is Present at Relatively High Concentrations	Use (Medicine, Dietary Supplements, Food)	Effects on the Endocannabinoid System	References
**Non-psychoactive cannabinoids**	**Cannabigerol (CBG)**	Antineoplastic	None demonstrated	All varieties	Food, medicine	Low affinity to the CB1 and CB2 receptors, and shows an ability to inhibit anandamide uptake	[16,27,32,43,44]
	**Cannabichromen (CBC)**	Antidepressant	None demonstrated	All varieties	Medicine	-	[27,32]
	**Cannabidiol (CBD)**	Analgesic, anti-inflammatory, anxiolytic, and antineoplastic	None demonstrated	All varieties, but mainly in *Cannabis sativa* L. var. *sativa*	Dietary supplements, food	Weak antagonistic action against the CB1 and CB2 receptors, and eliminates the effects of Δ9-THC	[31,44,45,46,47,48]
	**Δ9-Tetrahydrocannabivarin (Δ9-THCV),**	Treatment of obesity and epilepsy	None demonstrated	All varieties, but mainly in *Cannabis sativa* L. var. *sativa*	Medicine	Partial agonist of the CB2 receptors and antagonist of the CB1 receptors	[49,50]
**Psychoactive cannabinoids**	**Δ9-Tetrahydrocannabinol (Δ9-THC)**	Improves sleep and stimulates appetite in cancer patients	Demonstrated	All varieties, but mainly in *Cannabis sativa* L. var. *indica*	Medicine	Binds and activates the CB1 receptors	[31,51,52,53]
	**Δ8-Tetrahydrocannabinol (Δ8-THC)**	Anti-glaucoma, and supports the treatment of damaged epithelium of the cornea	Demonstrated, but weaker than Δ9-THC	All varieties, but mainly in *Cannabis sativa* L. var. *indica*	Medicine	Binds and activates the CB1 receptors	[54,55]
	**Cannabinol (CBN)**		Demonstrated, but 10× weaker than Δ9-THC	All varieties	-	Binds cannabinoid receptors, showing higher affinity to the CB2 receptors and weak agonism to the CB1 receptors	[25,44]

**Table 3:** Limitation of Δ9-THC content in food (ppm).

	Oil from Seeds	Seeds	Total Content in Food	References
**Germany** total content of Δ9-THC and Δ9-THCA	5	-	0.02–10	[79,80]
**Italy** total content of Δ9-THC and Δ9-THCA	5	2	2	[81]
**Switzerland** Δ9-THC	20	10	-	[82]
**Australia, New Zealand** total content of Δ9-THC and Δ9-THCA	10	5	<5	[83]
**Croatia** Δ9-THC	-	-	2–20	[84]
**Denmark** Δ9-THC	10	5	0.5	[85]

**Table 4:** Comparison of analytical methods for cannabinoids in food and beverages.

Matrix	Analytical Technique	Sample Preparation Method (Extraction, Purification)	Cannabinoids Determined	LOQ/LOD	References
Hemp oil	HPLC-UV HPLC-MS/MS	Extraction with 2-propanol	CBDA, Δ9-THCA, CBD, Δ9-THC, CBG, CBN, CBDV	1 mg/kg/0.2 mg/kg	[102]
Hemp oil	HPLC-HRMS	Extraction with 2-propanol	CBDV, CBDA, CBGA, CBG, CBD, CBN, Δ9-THC, Δ8-THC, CBC, Δ9-THCA	-	[129]
Hemp oil	GC-MS	Extraction with diethyl ether	CBD, CBN, Δ9-THC	0.03–0.1 mg/kg/-	[130]
Hemp oil	HPLC-Q-Exactive-Orbitrap-MS	-	CBD, Δ9-THC, CBN, CBG, CBDA, Δ9-THCA, CBGA	-	[45]
Beer, liqueur, seeds, oil hemp	GC-MS	Extraction with hexane/isopropanol mixture (9:1)	CBD, CBN, Δ9-THC	0.001–0.002/0.0003–0.0006 mg/kg	[131]
Hemp oil and commercially available consumer products (dietary supplements, food, candies, beverages)	HPLC-DAD	Extraction with 95% or 100% ethanol depending on the matrix type	CBD, CBDA, Δ9-THC, Δ9-THCA, CBN, Δ8-THC, CBG, CBGA, CBDV, Δ9-THCV, CBC	10 mg/kg/- (for all products)	[132]
Hemp oil and commercially available consumer products (among others: dietary supplements, food, candies, beverages)	GC-MS	Extraction with 95% or 100% ethanol depending on the matrix type	CBD, CBDA, Δ9-THC, Δ9-THCA, CBN, Δ8-THC, CBG, CBGA, CBDV, THCV, CBC	-/1 mg/kg (for all products)	[75]
Hemp oils Hemp-based extract	HPLC-UV/DAD	Extraction with isopropanol	CBG CBD	1.8 mg/kg/0.5 mg/kg 2.3 mg/kg/0.7 mg/kg	[133]
Hemp seeds, hemp protein	LC-MS/MS	Extraction with acetonitrile, QuEChERS	CBD, CBDA, Δ9-THC, Δ9-THCA, CBN, CBC, CBCA, CBDV, CBDVA, CBG, CBGA, THCV, THCVA, Δ8-THC	0.15 mg/kg/-	[39]
Hemp oil				0.6 mg/kg/-	
Raw and powdered milk				0.005 mg/kg/-	
Tea, coffee, chocolate				0.15 mg/kg/-	
Mayonnaise				0.006 mg/kg/-	
Food products	LC-MS/MS	Extraction with methanol:chloroform mixture (9:1, *v/v)*	Δ9-THC, THCA, Δ8 -THC, CBN, CBD, CBDA, CBG, CBGA THCV	0.02 mg/kg/0.006 mg/kg	[105]
Beverages		Extraction with methanol		0.002 mg/kg/0.6 mg/kg	
Milk	LC-MS/MS	Extraction with methanol, SPE	Δ9-THC, Δ9-THC-OH, Δ9-THCA	0.00413–0.00873 mg/kg/0.00444–0.00893 mg/kg	[134]
Hemp seeds				0.00310–0.00678 mg/kg/0.00352–0.00722 mg/kg	
Chocolate, energy bars, oils	LC-MS/MS	Filter only, SPE, dispersive-SPE, QuEChERS, EMR-lipid	Δ9-THC, CBD	0.00003 mg/kg/0.00001 mg/kg	[135]
Candies and jellies		Filter only, SPE, dispersive-SPE, QuEChERS			
Powdered hemp protein, snacks, and cereals					
Fermented mead with the addition of extracts from inflorescences, leaves, and stems	HPLC-FID	Extraction with hexane/ethyl acetate mixture (9:1 *v/v*)	CBD, CBN	-/0.01 mg/L	[136]
Tinctures and oils	HPLC-DAD	Extraction with 95% or 100% ethanol depending on the matrix type	CBDA, CBD, Δ9-THCA, Δ9-THC, Δ8 -THC, CBN, CBC, CBG, CBGA, CBDV, Δ9-THCV	1–10 mg/kg/4–40mg/kg (depending on the matrix)	[122]
Food products (honey, candies, jellies, cookies)					
Beverages					

## Data Availability

Data sharing not applicable.

## References

[1] Pellati F., Borgonetti V., Brighenti V., Biagi M., Benvenuti S., Corsi L. (2018). *Cannabis sativa* L. and non psychoactive cannabinoids: Their chemistry and role against oxidative stress, inflamation and cancer. BioMed Res. Int..

[2] Salami S.A., Martinelli F., Glovino A., Bachari A., Arad N., Mantri N. (2020). It is our turn to get *Cannabis* high: Put cannabinoids in food and health baskets. Molecules.

[3] Karas J.A., Wong L.J.M., Paulin O.K.A., Mazeh A.C., Hussein M.H., Li J., Velkov T. (2020). The antimicrobial activity of cannabinoids. Antibiotics.

[4] Baker D., Pryce G., Giovannoni G., Thompson A.J. (2003). The therapeutic potential of *Cannabis*. Lancet Neurol..

[5] Da Porto C., Decorti D., Natolino A. (2014). Potential oil yield, fatty acid composition, and oxidation stability of the hempseed oil from four *Cannabis sativa* L. cultivars. J. Diet. Suppl..

[6] Farinon B., Molinari R., Costantini L., Merendino N. (2020). The seed of industrial hemp (*Cannabis sativa* L.): Nutritional quality and potential functionality for human health and nutrition. Nutrients.

[7] Congressional Research Service: Hemp as an Agricultural Commodity (CRS Report RL32725). https://fas.org/sgp/crs/misc/RL32725.pdf.

[8] *Cannabis* Cultivation Market Size, Share & Trends Analysis Report by Biomass (Hemp, Marijuana), by Application (Medical Consumption, Recreational Consumption), by Region, and Segment Forecasts. (Repoert ID GVR-3-68038-803-9). https://www.grandviewresearch.com/industry-analysis/Cannabis-cultivation-market#.

[9] Hazekamp A., Fischedick J.T. (2012). *Cannabis*—From cultivar to chemovar. Drug Test. Anal..

[10] Soorni A., Fatahi R., Haak D.C., Salami S.A., Bombarely A. (2017). Assesment of Genetic Diversity and Population Strucutre in Iranian *Cannabis* Germplasm. Sci. Rep..

[11] André A., Leupin M., Kneubühl M., Pedan V., Chetschik I. (2020). Evolution of the polyphenol and terpene content, antioxidant activity and alant morphology of eight different fiber-type cultivars of *Cannabis sativa* L. cultivated at three sowing densities. Plants.

[12] Small E., Cronquist A. (1976). A practical and natural taxonomy for *Cannabis*. Taxon.

[13] Hartsel J.A., Eades J., Hickory B., Makriyannis A., Guptam C.R. (2016). Cannabis sativa and hemp. Nutraceuticals: Efficacy, Safety and Toxicity.

[14] Micalizzi G., Vento D., Alibrando F., Donnarumma D., Dugo P., Mondello L. (2021). *Cannabis sativa* L.: A comprehensive review on the analytical methodologies for cannabinoids and terpenes characterization. J. Chromatogr. A.

[15] Flores-Sanchez I.J., Verpoorte R. (2008). Secondary metabolism in *Cannabis*. Phytochem. Rev..

[16] ElSohly M.A., Radwan M.M., Gul W., Chandra S., Galal A., Kinghorn A., Falk H., Gibbons S., Kobayashi J. (2017). Phytochemistry of *Cannabis sativa* L.. Phytocannabinoids: Progress in the Chemistry of Organic Natural Products.

[17] Citti C., Braghiroli D., Vandelli M.A., Cannazza G. (2018). Pharmaceutical and biomedical analysis of cannabinoids: A critical review. J. Pharm. Biomed. Anal..

[18] Alonso-Esteban J.I., González-Fernández M.J., Fabrikov D., Torija-Isasa E., de Cortes Sánchez-Mata M., Guil-Guerrero J.L. (2020). Hemp (*Cannabis sativa* L.) varieties: Fatty acid profiles and upgrading of γ-linolenic acid–containing hemp seed oils. Eur. J. Lipid Sci. Technol..

[19] Bartkiene E., Schleining G., Krungleviviute V., Zadeike D., Zavistanaviciute P., Dimaite I., Kuzmaite L., Riskeviciene V., Juodeikiene G. (2016). Development and quality evaluation of lacto-fermented product based on hulled and not hulled hempseed (*Cannabis sativa* L.). LWT Food Sci. Technol..

[20] Spano M., Di Matteo G., Rapa M., Ciano C., Ingallina C., Cesa S., Menghini L., Carradori S., Giusti A.M., Di Sotto A. (2020). Commercial hemp seed oils: A multimethodological characterization. Appl. Sci..

[21] Pojić M., Dapčević Hadnađev T., Hadnađev M., Rakita S., Brlek T. (2015). Bread supplementation with hemp seed cake: A by-product of hemp oil processing. J. Food Qual..

[22] Leonard W., Zhang P., Ying D., Fang Z. (2019). Hempseed in food industry: Nutritional value, health benefits, and industrial applications. Compr. Rev. Food Sci. Food Saf..

[23] Callaway J.C. (2004). Hempseed as a nutritional resource: An overview. Euphytica.

[24] Casano S., Grassi G., Martini V., Michelozzi M. (2011). Variations in terpene profiles of different strains of *Cannabis sativa* L.. Acta Hortic..

[25] Tomko A.M., Whynot E.G., Ellis L.D., Dupré D.J. (2020). Anti-cancer potential of cannabinoids, terpenes and flavonoids present in *Cannabis*. Cancers.

[26] Ingallina C., Sobolev A.P., Circi S., Spano M., Fraschetti C., Filippi A., Di Sotto A., Di Giacomo S., Mazzoccanti G., Gasparrini F. (2020). *Cannabis sativa* L. inflorescences from monoecious cultivars grown in central Italy: An untargeted chemical characterization from early flowering to ripening. Molecules.

[27] Spano M., Di Matteo G., Ingallina C., Botta B., Quaglio D., Ghirga F., Balducci S., Cammarone S., Campiglia E., Giusti A.M. (2021). A multimethodological characterization of *Cannabis sativa* L. inflorescences from seven dioecious cultivars grown in Italy: The effect of different harvesting stages. Molecules.

[28] Wen W., Alseekh S., Fernie A.R. (2020). Conservation and diversification of flavonoid metabolism in the plant kingdom. Curr. Opin. Plant Biol..

[29] Horanin A., Bryndal I. (2017). Hemp—Active ingredients, medicinal properties and using. Res. Pap. Wroc. Univ. Econ..

[30] Carus M., Karst S., Kauffmann A., Hobson J., Bertucelli S. (2013). The European Hemp Industry: Cultivation, Processing and Applications for Fibres, Shives and Seeds.

[31] Gülck T., Møller B.L. (2020). Phytocannabinoids: Origins and biosynthesis. Trends Plant Sci..

[32] Ujváry I., Hanuš L. (2016). Human metabolites of cannabidiol: A review on their formation, biological activity, and relevance in therapy. Cannabis Cannabinoid Res..

[33] Hanuš L.O., Meyer S.M., Muñoz E., Taglialatela-Scafati G., Appendino G. (2016). Phytocannabinoids: A unified critical inventory. Nat. Prod. J..

[34] Nahar L., Guo M., Sarker S.D. (2019). Gas chromatographic analysis of naturally occurring cannabinoids: A review of literature published during the past decade. Phytochem. Anal..

[35] Radwan M.M., Chandra S., Gul S., ElSohly M.A. (2021). Cannabinoids, phenolics, terpenes and alkaloids of *Cannabis*. Molecules.

[36] Taura F., Tanaka S., Taguchu C., Fukamizu T., Tanaka H., Shoyama Y., Morimoto S. (2009). Characterization of olivetol synthase, a polyketide synthase putatively involved in cannabinoid biosynthetic pathway. FEBS Lett..

[37] Fellermeier M., Zenk M.H. (1998). Prenylation of olivetolate by a hemp transferase yields cannabigerolic acid, the precursor of tetrahydrocannabinol. FEBS Lett..

[38] Tahir N.M., Shahbazi F., Rondeau-Gagné S., Trant J.F. (2021). The biosynthesis of the cannabinoids. J. Cannabis Res..

[39] Christinat N., Savoy M.C., Mottier P. (2020). Development, validation and application of a LC-MS/MS method for quantification of 15 cannabinoids in food. Food Chem..

[40] Bonini S., Premoli M., Tambaro S., Kumar A., Maccarinelli G., Memo M., Mastinu A. (2018). *Cannabis sativa*: A comprehensive ethnopharmacological review of a medicinal plant with a long history. J. Ethnopharmacol..

[41] Palazzoli F., Citti C., Licata M., Vilella A., Manca L., Zoli M., Vandelli M.A., Forni F., Cannaza G. (2018). Development of a simple and sensitive liquid chromatography triple quadrupole mass spectrometry (LC–MS/MS) method for the determination of cannabidiol (CBD), Δ9-tetrahydrocannabinol (THC) and its metabolites in rat whole blood after oral administration of a single high dose of CBD. J. Pharm. Biomed. Anal..

[42] Mechoulam R., Shvo Y. (1963). The structure of cannabidiol. Tetrahedron.

[43] De Petrocellis L., Ligresti A., Schiano Moriello A., Iappelli M., Verde R., Stott C.G., Cristino L., Orlando P., Di Marzo V. (2013). Non-THC cannabinoids inhibit prostate carcinoma growth in vitro and in vivo: Pro-apoptotic effects and underlying mechanisms. Br. J. Pharmacol..

[44] Morales P., Hurst D.P., Reggio P.H. (2017). Molecular targets of the phytocannabinoids—A complex picture. Prog. Chem. Org. Nat. Prod..

[45] Pavlovic R., Nenna G., Calvi L., Panseri S., Borgonovo G., Giupponi L., Cannazza G., Giorgi A. (2018). Quality traits of “cannabidiol oils”: Cannabinoids content, terpene fingerprint and oxidation stability of European commercially available preparations. Molecules.

[46] Thomas A., Baillie G.L., Phillips A.M., Razdan R.K., Ross R.A., Pertwee R.G. (2007). Cannabidiol displays unexpectedly high potency as an antagonist of CB1 and CB2 receptor agonists in vitro. Br. J. Pharmacol..

[47] Afrin F., Chi M., Eamens A.L., Duchatel R.J., Douglas A.M., Schneider J., Gedye C., Woldu A.S., Dun M.D. (2020). Can hemp help? Low-THC *Cannabis* and non-THC cannabinoids for the treatment of cancer. Cancer.

[48] Kis B., Ifrim F.C., Buda V., Avram S., Pavel I.Z., Antal D., Paunescu V., Dehelean C.A., Ardelean F., Diaconeasa Z. (2019). Cannabidiol—from plant to human body: A promising bioactive molecule with milti-target effects in cancer. Int. J. Mol. Sci..

[49] Atakan Z. (2012). *Cannabis*, a complex plant: Different compounds and different effects on individuals. Ther. Adv. Psychopharmacol..

[50] Carrillo-Salinas F.J., Navarrete C., Mecha M., Feliu A., Collado J.A., Cantarero I., Bellido M.L., Muñoz E., Guaza C. (2014). A cannabigerol derivative suppresses immune responses and protects mice from experimental autoimmune encephalomyelitis. PLoS ONE.

[51] Pacher P., Batkai S., Kunos G. (2006). The endocannabinoid system as emerging target of pharmacotherapy. Pharmacol. Rev..

[52] Fine P.G., Rosenfeld M.J. (2013). The endocannabinoid system, cannabinoids, and pain. Rambam Maimonides Med. J..

[53] Chanda D., Neumann D., Glatz J.F.C. (2019). The endocannabinoid system: Overview of an emerging multi-faceted therapeutic target. Prostaglandins Leukot. Essent. Fat. Acids.

[54] Thapa D., Cairns E.A., Szczesniak A.M., Toguri J.T., Caldwell M.D., Kelly M.E.M. (2018). The cannabinoids D8THC, CBD, and HU-308 act via distinct receptors to reduce corneal pain and inflammation. Cannabis Cannabinoid Res..

[55] Vági E., Balázs M., Komóczi A., Kiss I., Mihalovits M., Székely E. (2019). Cannabinoids enriched extracts from industrial hemp residues. Period. Polytech. Chem. Eng..

[56] Gaoni Y., Mechoulam R. (1964). Isolation, structure, and partial synthesis of an active constituent of hashish. J. Am. Chem. Soc..

[57] Ramirez C.L., Fanovich M.A., Churio M.S. (2019). Cannabinoids: Extraction methods, analysis, and physicochemical characterization. Stud. Nat. Prod. Chem..

[58] Marcu J.P. (2016). An overview of major and minor phytocannabinoids. Neuropathol. Drug Addict. Subst. Misuse.

[59] Hippalgaonkar K., Gul W., ElSohly M.A., Repka M.A., Majumdar S. (2011). Enhanced solubility, stability, and transcorneal permeability of δ-8-tetrahydrocannabinol in the presence of cyclodextrins. AAPS PharmSciTech.

[60] Rosenthaler S., Pöhn B., Kolmanz C., Huu C.N., Krewenka C., Huber A., Kranner B., Rausch W.D., Moldzio R. (2014). Differences in receptor binding affinity of several phytocannabinoids do not explain their effects on neural cell cultures. Neurotoxicol. Teratol..

[61] Citti C., Linciano P., Russo F., Luongo L., Iannotta M., Maione S., Laganà A., Capriotti A.L., Forni F., Vandelli M.A. (2019). A novel phytocannabinoid isolated from *Cannabis sativa* L. with an in vivo cannabimimetic activity higher than Δ9-tetrahydrocannabinol: Δ9-Tetrahydrocannabiphorol. Sci. Rep..

[62] Appendino G., Gibbons S., Giana A., Pagani A., Grassi G., Stavri M., Smith E., Rahman M.M. (2008). Antibacterial cannabinoids from *Cannabis sativa*: A structure-activity study. J. Nat. Prod..

[63] Ali E.M.M., Almagboul A.Z.I., Khogali S.M.E., Gergeir U.M.A. (2012). Antimicrobial activity of *Cannabis sativa* L.. Chin. Med..

[64] Lone T.A., Lone R.A. (2012). Extraction of cannabinoids from *Cannabis sativa* L. plant and its potential antimicrobial activity. Univers. J. Med. Dent..

[65] Iseppi R., Brighenti W., Licata M., Lambertini A., Sabia C., Messi P., Pellati F., Benvenuti S. (2019). Chemical characterization and evaluation of the antibacterial activity of essential oils from fibre-type *Cannabis sativa* L. (hemp). Molecules.

[66] Frassinetti S., Gabriele M., Moccia E., Longo V., Di Giola D. (2020). Antimicrobial and antibiofilm activity of *Cannabis sativa* L. seeds extract against *Staphylococcus aureus* and growth effects on probiotic *Lactobacillus* spp.. LWT Food Sci. Technol..

[67] Huestis M.A., Pertwee G.R. (2005). Pharmacokinetics and metabolism of the plant cannabinoids, ∆9-tetrahydrocannabinol. Cannabinoids.

[68] Garrett E.R., Hunt C.A. (1974). Physicochemical properties, solubility, and protein binding of ∆9-tetrahydrocannabinol. J. Pharm. Sci..

[69] Gonçalves J., Rosado T., Soares S., Simão A.Y., Caramelo D., Luís Â., Fernández N., Barroso M., Gallardo E., Duarte A.P. (2019). *Cannabis* and its secondary metabolites: Their use as therapeutic drugs, toxicological aspects, and analytical determination. Medicines.

[70] Grotenhermen F. (2003). Pharmacokinetics and pharmacodynamics of cannabinoids. Clin. Pharmacokinet..

[71] Lucas C.J., Galettis P., Schneider J. (2018). The pharmacokinetic and the pharmacodynamics of cannabinoids. Br. J. Clin. Pharmacol..

[72] Grant K.S., Petroff R., Isoherranen N., Stella N., Burbacher T.M. (2018). *Cannabis* use during pregnancy: Pharmacokinetics and effects on child development. Pharmacol. Ther..

[73] Ahmed A.I.A., van der Elsen G.A.H., Colbers A., Kramers C., Burger D.M., van der Marck M.A., Olde Rikkert M.G.M. (2015). Safety, pharmacodynamics, and pharmacokinetics of multiple oral doses of delta-9-tetrahydrocannabinol in older persons with dementia. Psychopharmacology.

[74] Degenhardt L., Hall W. (2012). Extent of illicit drug use and dependence, and their contribution to the global burden of disease. Lancet.

[75] Ciolino L.A., Ranieri T.L., Taylor A.M. (2018). Commercial *Cannabis* consumer products part 1: GC-MS quantitative analysis of *Cannabis* cannabinoids. Forensic Sci. Int..

[76] Lachenmeier D.W., Walch S.G. (2005). Analysis and toxicological evaluation of cannabinoids in hemp food products—A review. Electron. J. Environ. Agric. Food Chem..

[77] Fischedick J.T., Hazekamp A., Erkelens T., Choi Y.H., Verpoorte R. (2010). Metabolic fingerprinting of *Cannabis sativa* L., cannabinoids and terpenoids for chemotaxonomic and drug standardization purposes. Phytochemistry.

[78] Aizpurua-Olaizola O., Omar J., Navarro P., Olivares M., Etxebarria N., Usobiaga A. (2014). Identification and quantification of cannabinoids in *Cannabis sativa* L. plants by high performance liquid chromatography-mass spectrometry. Anal. Bioanal. Chem..

[79] European Industrial Hemp Association Reasonable Guidance Values for THC (Tetrahydrocannabinol) in Food Products. http://eiha.org/media/2017/09/17-09-18-THC-Position-paper_EIHA.pdf.

[80] BgVV Recommends Guidance Values for THC (Tetrahydrocannabinol) in Hemp-Containing Foods (07/2000). http://www.bfr.bund.de/en/presseinformation/2000/07/bgvv_recommends_guidance_values_for_thc__tetrahydrocannabinol__in_hemp_containing_foods-1309.html.

[81] United States Department of Agriculture Foreign Agricultural Service: Italian industrial hemp overview (Report No. IT2020-0001). https://apps.fas.usda.gov/newgainapi/api/Report/DownloadReportByFileName?fileName=Italian%20Industrial%20Hemp%20Overview%202020%20_Rome_Italy_02-18-2020.

[82] European Industrial Hemp Association (EIHA) Evaluation of Limit and Guideline Values of THC (Tetrahydrocannabinol) in Hemp Foods. https://eiha.org/media/2019/06/19-06-18_Limit-and-guideline-values-for-THC-in-hempfoods.pdf.

[83] Food Standards Australia New Zealand (FSANZ) (2017). Australia New Zealand Food Standards Code—Standard 1.4.4—Prohibited and Restricted Plants and Fungi (No. F2016C00169). https://www.legislation.gov.au/Details/F2017C01047/Download.

[84] Croatian Food Agency (HAH) (2011). Scientific Opinion on the Health Effects of Hemp Products Consumed (Oil, Seeds) (HAH Publication No. HAH-Z-2011-4). https://www.hah.hr/znanstveno-misljenje-o-utjecaju-na-zdravlje-proizvoda-od-konoplje-koji-se-konzumiraju-ulje-sjemenke/.

[85] Danish Veterinary and Food Administration (DVFA) (2018). Guidance Levels for Tetrahydrocannabinol Content in Foodstuffs from Industrial Hemp. https://www.foedevarestyrelsen.dk/SiteCollectionDocuments/Kemi%20og%20foedevarekvalitet/GMONovel%20foodNanoBestraaling/Information%20on%20webpage%20about%20guidance%20levels%20for%20THC%20in%20hemp_EN.pdf.

[86] Regulation (EU) 1307/2013. https://eur-lex.europa.eu/LexUriServ/LexUriServ.do?uri=OJ:L:2013:347:0608:0670:en:PDF.

[87] Regulation (EU) 2015/2283. https://eur-lex.europa.eu/legal-content/EN/TXT/PDF/?uri=CELEX:32015R2283&from=EN.

[88] European Food Safety Authority (EFSA) (2015). Scientific Opinion on the risks for human health related to the presence of tetrahydrocannabinol (THC) in milk and other food of animal origin. Front. Pharmacol..

[89] Commision Recommendation EU (2016/2115). https://eur-lex.europa.eu/legal-content/EN/TXT/PDF/?uri=CELEX:32016H2115&from=PLp.

[90] Montoya Z., Conroy M., Heuvel B.D.V., Pauli C.S., Park S.H. (2020). *Cannabis* contaminant limit pharmacological use of cannabidiol. Front. Pharmacol..

[91] Bonn-Miller M.O., Loflin M.J.E., Thomas B.F., Marcu J.P., Hyke T., Vandrey R. (2017). Labeling accuracy of cannabidiol extracts sold online. JAMA.

[92] Lachenmeier D.W., Habel S., Fischer B., Herbi F., Zerbe T., Bock V., Rajcic de Rezende T., Walch S.G., Sproll C. (2020). Are side effects of cannabidiol (CBD) products caused by tetrahydrocannabinol (THC) contamination?. F1000Research.

[93] Barrus D.G., Capogrossi K.L., Cates S.C., Gourdet C.K., Peiper C.N., Novak S.P., Lefever T.W., Wiley J.L. (2016). Tasty THC: Promises and Challenges of *Cannabis* Edibles. Methods Rep. RTI Press.

[94] Sun-Waterhouse D., Penin-Peyta L., Wdhwa S.S., Waterhouse G.I.N. (2012). Storage stability of phenolic-fortified avocado oil encapsulated using different polymer formulations and co-extrusion technology. Food Bioprocess. Technol..

[95] Ruiz Ruiz J.C., Ortiz Vazques E.D.L.L., Segura Campos M.R. (2017). Encapsulation of vegetable oils as source of omega-3 fatty acids for enriched functional foods. Crit. Rev. Food Sci. Nutr..

[96] Chen P., Rogers M.A. (2019). Opportunities and challenges in developing orally-administered *Cannabis* edibles. Curr. Opin. Food Sci..

[97] Teo A., Dimartino S., Lee S.J., Goh K.K.T., Wen J., Oey I., Ko S., Kwakm H.S. (2016). Interfacial structures of whey protein isolate (WPI) and lactoferrin on hydrophobic surfaces in a model system monitored by quartz crystal microbalance with dissipation (QCM-D) and their formation on nanoemulsions. Food Hydrocoll..

[98] Charoen R., Jangchud A., Jangchud K., Harnsilawat T., Naivikul O., McClements D.J. (2011). Influence of biopolymer emulsifier type on formation and stability of rice bran oil-in-water emulsions: Whey protein, gum arabic, and modified starch. J. Food Sci..

[99] Ozturk B., Argin S., Ozilgen M., McClements D.J. (2014). Formation and stabilization of nanoemulsion-based vitamin E delivery systems using natural surfactants: Quillaja saponin and lecithin. J. Food Eng..

[100] Rasera G.B., Ohara A., Soares de Castro R.J. (2021). Innovative and emerging applications of *Cannabis* in food and beverage products: From an illicit drug to a potential ingredient for health promotion. Trends Food Sci. Technol..

[101] Rupasinghe H.P.V., Davis A., Kumar K.S., Murray B., Zheljazkov V.D. (2020). Industrial hemp (*Cannabis sativa* subsp. *sativa*) as an emerging source for value-added functional food ingredients and nutraceuticals. Molecules.

[102] Citti C., Pacchetti B., Vandelli M.A., Forni F., Cannazza G. (2018). Analysis of cannabinoids in commercial hemp seed oil and decarboxylation kinetics studies of cannabidiolic acid (CBDA). J. Pharm. Biomed. Anal..

[103] Formato M., Crescente G., Scognamiglio M., Fiorentino A., Pecoraro M.T., Piccolella S., Catauro M., Pacifico S. (2020). (‒)-cannabidiolic acid, a still overlooked bioactive compound: An introductory review and preliminary research. Molecules.

[104] Thomas B.F., ElSohly M.A. (2015). Biosynthesis and Pharmacology of Phytocannabinoids and Related Chemical Contituents. The Analytical Chemistry of Cannabis: Quality Assessment, Assurance, and Regulation of Medicinal Marijuana and Cannabinoid Preparations.

[105] Pisciottano I.M., Guadagnuolo G., Soprano V., Esposito M., Gallo P. (2021). A survey of Δ9-THC and relevant cannabinoids in products from the Italian market: A study by LC–MS/MS of food, beverages and feed. Food Chem..

[106] House J.D., Neufeld J., Leson G. (2010). Evaluating the quality of protein from hemp seed (*Cannabis sativa* L.) products through the use of the protein digestibility-corrected amino acid score method. J. Agric. Food Chem..

[107] Montserrat-de la Paz S., Marin-Aguilar F., Garcia-Giménez M.D., Fernández-Arche M.A. (2014). Hemp (*Cannabis sativa* L.) seed oil: Analytical and phytochemical characterization of the unsaponifiable fraction. J. Agric. Food Chem..

[108] Steinbach W. (1999). Hemp Pralines. DE Patent.

[109] Shim J.S. (2019). Manufacturing Method of Bread Containing Blue Ginseng Seed. KR Patent.

[110] Guang H., Wenwei C. (2006). Application of powder of whole Cannabis sativa seeds for preparing functional food with adjuvant therapy of lowering blood fat. China Patent.

[111] Berghofer E., Pollmann K., Traby M., Frenkenberger C. (2012). Method for Producing Hemp Milk. CA Patent.

[112] Bisterfeld von Merr G. (2014). Method of Obtaining Hemp Plant Juice and Use of Same for the Production of Beverages. U.S. Patent.

[113] Carvalho Â., Halkjær Hansen E., Kayser O., Carlsen S., Stehle F. (2017). Desinging microorganisms for heterologous biosynthesis of cannabinoids. FEMS Yeast Res..

[114] Dussy F.E., Hamberg C., Luginbühl M., Schwerzmann T., Briellmann T.A. (2005). Isolation of Δ9-THCA-A from hemp and analytical aspects concerning the determination of Δ9-THC in *Cannabis* products. Forensic Sci. Int..

[115] Fodor B., Molnár-Perl I. (2017). The role of derivatization techniques in the analysis of plant cannabinoids by gas chromatography mass spectrometry. TrAC Trends Anal. Chem..

[116] Radwan M.M., Wanas A.S., Chandra S., ElSholy M.A., Chandra S., Lata H., ElSohly M. (2017). Natural cannabinoids of *Cannabis* and methods of analysis. Cannabis sativa L.: Botany and Biotechnology.

[117] Berman P., Futoran K., Lewitus G.M., Mukha D., Benami M., Shlomi T., Meiri D. (2018). A new ESI-LC/MS approach for comprehensive metabolic profiling of phytocannabinoids in *Cannabis*. Sci. Rep..

[118] McRae G., Melanson J.E. (2020). Quantitative determination and validation of 17 cannabinoids in *Cannabis* and hemp using liquid chromatography-tandem mass spectrometry. Anal. Bioanal. Chem..

[119] De Backer B., Debrus B., Lebrun P., Theunis L., Dubois N., Decock L., Verstraete A., Hubert P., Charlier C. (2009). Innovative development and validation of an HPLC/DAD method for the qualitative and quantitative determination of major cannabinoids in *Cannabis* plant material. J. Chromatogr. B.

[120] Gul W., Gul S.W., Radwan M.M., Wanas A.S., Khan I.I., Sharaf M.H., ElSohly M.A. (2015). Determination of 11 cannabinoids in biomass and extracts of different varieties of *Cannabis* using high-performance liquid chromatography. J. AOAC Int..

[121] Dubrow G.A., Pawar R.S., Srigley C., Fong Sam J., Talavera C., Parker C.H., Noonan G.O. (2021). A survey of cannabinoids in hemp-derived products from the United States marketplace. J. Food Compos. Anal..

[122] Marchetti L., Brighenti V., Rossi M.C., Sperlea J., Pellati F., Bertelli D. (2019). Use of 13C-qNMR Spectroscopy for the analysis of non-psychoactive cannabinoids in fibre-type *Cannabis sativa* L. (hemp). Molecules.

[123] Smith R.N. (1975). High-pressure liquid chromatography of *Cannabis*. Identification of separated constituents. J. Chromatogr. A.

[124] Mandrioli M., Tura M., Scotti S., Toschi T.G. (2019). Fast detection of 10 cannabinoids by RP-HPLC-UV method in *Cannabis sativa* L.. Molecules.

[125] Žampachová L., Aturki Z., Mariani F., Bednář P. (2021). A rapid nano-liquid chromatographic method for the analysis of cannabinoids in *Cannabis sativa* L. Extracts. Molecules.

[126] Glivar T., Eržen J., Kreft S., Zagožen M., Čerenak A., Čeh B., Tavčar Benković E. (2020). Cannabinoid content in industrial hemp (*Cannabis sativa* L.) varieties grown in Slovenia. Ind. Crop. Prod..

[127] Cardenia V., Toschi T.G., Scappini S., Rubino R.C., Rodriguez-Estrada M.T. (2018). Development and validation of a fast gas chromatography/mass spectrometry method for the determination of cannabinoids in *Cannabis sativa* L.. J. Food Drug Anal..

[128] Jang E., Kim H., Jang S., Lee J., Baeck S., In S., Kim E., Kim Y., Han E. (2020). Concentrations of THC, CBD, and CBN in commercial hemp seeds and hempseed oil sold in Korea. Forensic Sci. Int..

[129] Citti C., Linciano P., Panseri S., Vezzalini F., Forni F., Vandelli M.A., Cannazza G. (2019). Cannabinoid profiling of hemp seed oil by liquid chromatography coupled to high-resolution mass spectrometry. Front. Plant Sci..

[130] Fernández N., Carreras L.J., Larcher R.A., Ridolfi A.S., Quiroga P.N. (2020). Quantification of cannabinoids in *Cannabis* oil using GC/MS: Method development, validation, and application to commercially available preparations in Argentina. Planta Med. Int. Open.

[131] Pellegrini M., Marchei E., Pacifici R., Pichini S. (2005). A rapid and simple procedure for the determination of cannabinoids in hemp food products by gas chromatography-mass spectrometry. J. Pharm. Biomed. Anal..

[132] Ciolino L.A., Ranieri T.L., Taylor A.M. (2018). Commercial *Cannabis* consumer products part 2: HPLC-DAD quantitative analysis of *Cannabis* cannabinoids. Forensic Sci. Int..

[133] Brighenti V., Pellati F., Steinbach M., Meran D., Benvenuti S. (2017). Development of a new extraction technique and HPLC method for the analysis of non-psychoactive cannabinoids in fibre-type *Cannabis sativa* L. (hemp). J. Pharm. Biomed. Anal..

[134] Escrivá Ú., Andrés-Costa M.J., Andreu V., Picó Y. (2017). Analysis of cannabinoids by liquid chromatography-mass spectrometry in milk, liver and hemp seed to ensure food safety. Food Chem..

[135] Lee J.H., Min M.A.Y., Han J.H., Yang Y.J., Kim H., Shin D. (2020). Development and validation of LC-MS/MS method with QuEChERS cleanup for detecting cannabinoids in foods and dietary supplements. Food Addit. Contam. Part A.

[136] Romano R., Aiello A., De Luca L., Sica R., Caprio E., Pizzolongo F., Blaiotta G. (2021). Characterization of a new type of mead fermented with *Cannabis sativa* L. (hemp). J. Food Sci..

[137] Crippa J.A., Crippa A.C., Hallak J.E., Martín-Santos R., Zuardi A.W. (2016). Δ9-THC Intoxication by cannabidiol enriched *Cannabis* extract in two children with refractory epilepsy: Full remission after switching to purified cannabidiol. Front. Pharmacol..

[138] Lazarjani M.P., Torres S., Hooker T., Fowlie C., Young O., Seyfoddin A. (2020). Methods for quantification of cannabinoids: A narrative review. J. Cannabis Res..

[139] Lindholst C. (2010). Long term of stability *Cannabis* resin and *Cannabis* extracts. Aust. J. Forensic Sci..

[140] King J.W. (2019). The relationship between *Cannabis*/hemp use in foods and processing methodology. Curr. Opin. Food Sci..

[141] Fairbarin J.W., Liebmann J.A., Rowan M.G. (1976). The stability of *Cannabis* and its preparations on storage. J. Pharm. Pharmacol..

[142] Grafström K., Andersson K., Pettersson N., Dalgaard J., Dunne S.J. (2019). Effects of long term storage on secondary metabolite profiles of *Cannabis* resin. Forensic Sci. Int..

[143] Trofin I.G., Dabija G., Vaireanu D.I., Filipescu L. (2012). The influence of long-term storage conditions on the stability of cannabinoids derived from *Cannabis* resin. Rev. Chim..

[144] Trofin I.G., Dabija G., Vaireanu D.I., Filipescu L. (2012). Long-term storage and *Cannabis* oil stability. Rev. Chim..

[145] Meija J., McRae G., Miles C.O., Melanson J.E. (2021). Thermal stability of cannabinoids in dried *Cannabis*: Kinetic study. Anal. Bioanal. Chem..

[146] Zamengo L., Bettin C., Badocco D., Marco V.D., Miolo G., Frison G. (2019). The role of time and storage conditions on the composition of hashish and marijuana samples: A four-year study. Forensic Sci. Int..

[147] Peschel W. (2016). Quality control of traditional *Cannabis* tinctures: Pattern, markers and stability. Sci. Pharm..

[148] Pacifici R., Marchel E., Salvatore F., Guandalini L., Busardò O., Pichini S. (2017). Evaluation of cannabinoids concentration and stability in standardized preparations of *Cannabis* tea and *Cannabis* oil by ultra-high performance liquid chromatography tandem mass spectrometry. Clin. Chem. Lab. Med..

[149] Milay L., Berman P., Shapira A., Guberman O., Meiri D. (2020). Metabolic profiling of *Cannabis* secondary metabolites for evaluation of optimal postharvest storage conditions. Front. Plant Sci..

[150] Mudge E.M., Murch S.J., Brown P.N. (2017). Leaner and greener analysis of cannabinoids. Anal. Bioanal. Chem..

[151] Turek C., Florian C.S. (2013). Stability of essential oils: A review. Compr. Rev. Food Sci. Food Saf..

[152] Zhong Y., Shahidi F. (2010). Lipid oxidation and improving the oxidative stability. Chem. Soc. Rev..

[153] Singh A.P., Fathordoobady F., Guo Y., Singh A., Kitts D.D. (2020). Antioxidants help favorably regulate the kinetics of lipid peroxidation, polyunsaturated fatty acids degradation and acidic cannabinoids decarboxylation in hempseed oil. Sci. Rep..

[154] Wang M., Wang Y.H., Avula B., Radwan M.M., Wanas A.S., van Antwerp J., Parcher J.F., ElSohly S.A., Khan I.A. (2016). Decarboxylation study of acidic cannabinoids: A novel approach using ultra-high-performance supercritical fluid chromatography/photodiode array-mass spectrometry. Cannabis Cannabinoid Res..

[155] Casiraghi A., Roda G., Casagni E., Cristina C., Musazzi U.M., Franzè S., Rocco P., Giuliani C., Fico G., Minghetti P. (2017). Extraction method and analysis of cannabinoids in *Cannabis* olive oil preparations. Planta Medica.

[156] Taschwer M., Schmid M.G. (2015). Determination of the relative percentage distribution of THCA and Δ9-THC in herbal *Cannabis* seized in Austria—Impact of different storage temperatures on stability. Forensic Sci. Int..

[157] Knezevic F., Nikolai A., Marchart R., Sosa S., Tubaro A., Novak J. (2021). Residues of herbal hemp leaf teas—How much of the cannabinoids remain?. Food Control.

[158] Ryu B.R., Islam M.J., Azad M.O.K., Go E.-J., Rahman M.H., Rana M.S., Lim Y.-S., Lim J.D. (2021). Conversion characteristics of some major cannabinoids from hemp (*Cannabis sativa* L.) raw. materials by new rapid simultaneous analysis method. Molecules.

[159] Dawson D.D., Martin R.W. (2020). Investigation of chocolate matrix interference on cannabinoid analytes. J. Agric. Food Chem..

[160] Wolf C.E., Poklis J.L., Poklis A. (2017). Stability of tetrahydrocannbinol and cannabidiol in prepared quality control medible brownies. J. Anal. Toxicol..

